# Orthogonal Investigation at Single-Particle and Ensemble Levels Uncovers Lipoprotein-Extracellular Vesicle Binding

**DOI:** 10.1021/acs.analchem.5c05327

**Published:** 2026-01-08

**Authors:** Angelo Musicò, Roberto Frigerio, Karl Normak, Sabrina Scolari, Alessandro Gori, Paolo Arosio, Annalisa Radeghieri, Lucia Paolini, Miriam Romano, Irantzu Llarena, Sergio E. Moya, Andrea Zendrini, Paolo Bergese

**Affiliations:** † Department of Molecular and Translational Medicine (DMMT), 9297 University of Brescia, 25123 Brescia, Italy; ‡ Istituto di Scienze e Tecnologie Chimiche ‘Giulio Natta’’—National Research Council of Italy (SCITEC−CNR), 20131 Milan, Italy; § Department of Chemistry and Applied Biosciences, Institute for Chemical and Bioengineering, 536519 ETH Zürich, 8093 Zürich, Switzerland; ∥ Center for Colloid and Surface Science (CSGI), 50019 Sesto Fiorentino, Italy; ⊥ Department of Medical and Surgical Specialties, Radiological Sciences and Public Health (DSMC), University of Brescia, 25123 Brescia, Italy; # ASST Spedali Civili di Brescia, Department of Emergency, Spedali Civili University Hospital, 25123 Brescia, Italy; ∇ Soft Matter Nanotechnology, Center for Cooperative Research in Biomaterials (CIC biomaGUNE), Basque Research and Technology Alliance (BRTA), 20850 Donostia-San Sebastian, Spain; ○ Faculty of Medicine, Chulalongkorn University, 10330 Bangkok, Thailand; ◆ National Inter-University Consortium of Materials Science and Technology (INSTM), 50121 Firenze, Italy

## Abstract

Mesoscale interactions critically shape the biological identity of extracellular nanoparticles, including extracellular vesicles. These interactions encompass biomolecular coronas, transient aggregation, and fusion events. Among them, the interaction between extracellular vesicles and lipoproteins has recently garnered significant attention due to their potential impact on functionality and in vivo fate of extracellular vesicles. In this work, we present a first investigation of the binding between human red blood cell-derived extracellular vesicles and lipoproteins across multiple scales, in both buffer and plasma. Red blood cell-derived extracellular vesicles were selected as a model system for their physicochemical homogeneity, potential in personalized medicine, and production scalability. To achieve this, we employed an ad hoc suite of orthogonal analytical techniques: fluorescence cross-correlation spectroscopy (FCCS), super-resolution microscopy, flow cytometry, and Single Molecule Array assays (Simoa). Our results reveal class-specific and context-dependent extracellular vesicle–lipoprotein associations. Notably, lipoproteins bind to extracellular vesicles with affinities ranging from 10 nM to 1 μM and with up to 100% extracellular vesicles interacting with high-density lipoproteins in the presence of plasma proteins. These findings uncover a complex and dynamic interactome of red blood cell-derived extracellular vesicles across lipoprotein classes. This work establishes a robust methodological framework for studying mesoscale interactions of extracellular nanoparticles under physiologically relevant conditions. Its versatility allows for its application to diverse interaction scenarios, supporting systematic investigation of context-dependent effects on EV–LP binding.

## Graphical abstract

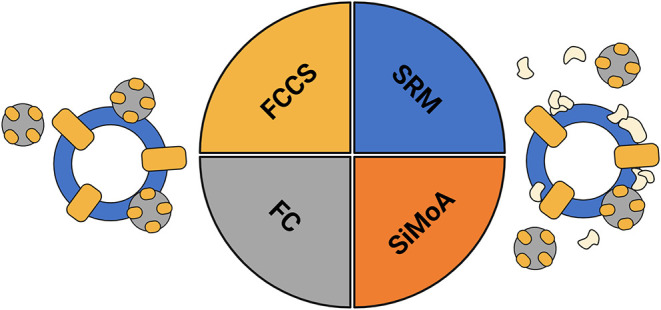

## Introduction

Biological fluids are nanostructured and populated with various types of extracellular nanoparticles (ENPs), which solve fundamental roles in their host organisms.^1^ Among the various ENPs, lipoproteins (LPs) and extracellular vesicles (EVs) stand out due to both the significant interest they have garnered from the scientific community and their strong translational potential in biomedicine. LPs are micelle-like nanoparticles that facilitate the transport of nonpolar lipids through the polar environment of the bloodstream, delivering them to cells and tissues where lipids are processed or stored. EVs comprise a diverse population of lipid membrane-bound vesicles, whose natural ability to transport and deliver bioactive cargo makes them key regulators of essential physio- and pathological processes.^2^ From a thermodynamic perspective, biological fluids—like any other nanostructured system—exhibit a high surface-to-volume ratio, mirrored by an increase in the free surface energy of ENPs. This, combined with crowding effects, drives the formation of a network of interactions among ENPs, which spontaneously and dynamically associate to relieve excess energy.^3–6^ A key example of these interactions is the biomolecular corona (BC).^7–11^ Initially introduced in the context of synthetic nanoparticles,^12,13^ this concept has since been extended to ENPs, in particular to EVs. BC refers to the dynamic coating of biomolecules (or other ENPs) that forms on the surface of nanomaterials when immersed in a biological fluid. The BC can be considered an ’extrinsic and contextual’ characteristic of the EVs, as it depends not only on EV traits, but also on the surrounding environment.^14–16^ Ultimately, these spatially correlated and time-resolved interactions are revealed to play a key role in shaping EV functions and, in some cases, to be mandatory for the biological activity of EVs.^10,17–20^ Indeed, the BC contributes to the EV cellular uptake, the activation of specific cellular responses, as well as to EV clearance and biodistribution in the organism.^21^ Defining a precise boundary between the EVs and the interacting molecular, nano- or micron-sized elements of the biological environment may not be straightforward. Recent thermodynamic considerations suggest that the energy required to form and maintain the structure of an EV is comparable to that involved in the nonspecific adsorption of a few dozen proteins onto its surface.^15^ This not only presents significant challenges in isolating pure EVs without altering their structure, composition, and ultimately function^22,23^ but also suggests that such “EV interactome” might be considered part of the EVs, and one of their constitutive traits. LPs have been traditionally considered mostly as coisolated, noninteracting contaminants in plasma EV preparations. However, recent findings suggest that EVs and LPs physically associate, exchanging material, fusing, and forming complexes. EV–LP interactions might be favored by their physiological ratio, which is heavily skewed toward LPs. Depending on the LP subtype, LPs in blood plasma outnumber EVs by as much as 100000:1.^24^ This imbalance, together with specific molecular recognition, may enhance the likelihood of association or fusion events, which to date seem to play functional roles in biological systems. For instance, lipoprotein levels in cell culture medium influence the effects of EVs on recipient cells *in vitro*, likely due to binding and fusion phenomena occurring between EVs and LPs. Similar interactions have also been observed in plasma.^25^ Additionally, EVs produced by brain-tropic breast cancer cells aggregate with low-density lipoproteins (LDL). This type of interaction has been shown to enhance the delivery of nanostructured drug carriers across the blood-brain barrier and may, therefore, have broader implications for targeted EV-mediated metastasis to the brain.^26^ Similarly, ApoE-containing LPs produced by hepatocytes accumulate within CD63-positive vesicles,^27^ and ApoE-carrying EVs have been shown to modify the lipid synthesis of hepatocytes *in vitro*.^28^ Finally, the formation of EV–LP complexes may also influence the pharmacokinetics and biodistribution of EVs,^29^ potentially impacting their effectiveness as natural drug delivery systems, either positively or negatively. This aspect is, for instance, already reported for synthetic lipid-based drug nanocarriers,^30–32^ and has also been exploited to enhance their delivery to specific organs.^33–35^ Investigating EV–LP interactions in a native biological environment remains highly challenging due to the inherent complexity and inaccessibility of the media. To overcome these limitations, we developed a bottom-up methodology, schematized in Figure 1. Our approach utilizes a standardized model system that combines extracellular vesicles with narrow physicochemical properties and purified commercial lipoproteins in buffer and in a close physiological environment as possible. The characterization of EV–LP interactions in pure buffer was included to reproduce conditions like those commonly used in model nanomaterial-biomolecule interaction systems,^36,37^ thus establishing a baseline for such an interaction. This preliminary step allows us to discriminate intrinsic EV–LP binding properties from the additional contributions of plasma proteins. Therefore, even if incubation in pure buffer does not reproduce physiological conditions it provides essential reference information to assess how plasma components modulate or shift the equilibrium of the interaction.^38^ We first mixed red blood cell-derived EVs (REVs) with representative classes of purified lipoproteins in buffer, at physiological ratios, then we added defined amounts of human plasma that had been depleted of extracellular nanoparticles. REVs have been chosen because they bear a unique balance of physicochemical and biological characteristics, bioprocessing, and scaling-up advantages, making them suitable for model experiments, and they are highly appealing candidates as next-generation nanoplatforms for nanomedicine.^6,11,39^ The classes of lipoproteins include high-density lipoprotein (HDL), low-density lipoprotein (LDL), and very-low-density lipoprotein (VLDL), which have been suggested in previous studies to exhibit significant binding with EVs.^5,25,26^ To minimize interference and capture these interactions with precision, we implemented a complementary set of specialized analytical and orthogonal tools, operating at both the single-particle and ensemble levels. This synergistic strategy enables obtaining information *in situ* and under physiological conditions, with each technique operating on different chemical or physical principles to provide insight into EV–LP interactions at different analytical levels, allowing for qualitative cross-comparison of the results to further inform on the nature and extent of EV–LP interactions. The tools include Super-Resolution Microscopy (SRM, specifically direct Stochastic Optical Reconstruction Microscopy, dSTORM), Flow Cytometry (FC), Fluorescence Cross-Correlation Spectroscopy (FCCS), and specifically developed Single Molecule Array (SiMoA) assays, complemented by standard biochemical and biophysical techniques used for the characterization of nanomaterials.

**Figure 1:**
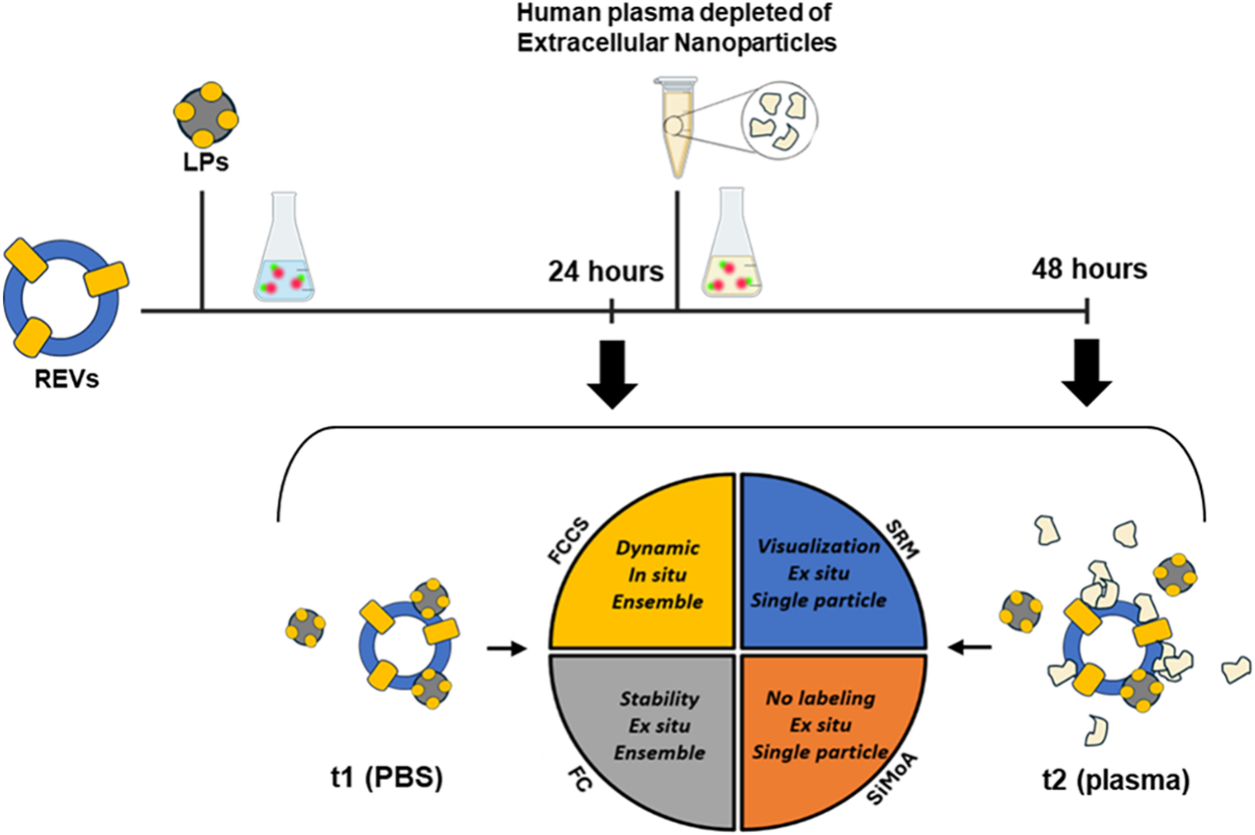
Schematic representation of the concept and methodology used to study the interaction between EVs and LPs under near-to-physiological conditions. The top section illustrates the timeline of the bottom-up approach, leading to the preparation of t1 (overnight incubation in PBS) and t2 (an additional 24-h incubation with the addition of human plasma depleted of extracellular nanoparticles) samples. A graphical representation of the readout and main features for each of the techniques used is also given in the lower part.

## Materials and Methods

### REV Extraction and Purification

Blood samples were collected from healthy donors at Spedali Civili Hospital in Brescia, Italy. All experiments involving blood samples were conducted in accordance with current guidelines and were approved by the Spedali Civili Ethics Committee. Red blood cells (RBCs) were obtained from anonymized healthy volunteers under written informed consent and provided by A.O. Spedali Civili di Brescia (ethical approval: “EritrEV NP5705”). Red blood cell-derived extracellular vesicles (REVs) were isolated under sterile conditions following the protocol developed by Usman et al., which describes a cost-effective method for purifying large quantities of EVs from RBCs. Briefly, concentrated RBCs were separated by centrifugation at 1000*g* for 8 min at 4 °C and washed three times with phosphate-buffered saline (PBS). The cells were then washed twice more with PBS containing 0.1 g/L calcium chloride (CPBS) and transferred to a 75 cm^2^ tissue culture flask. RBCs were incubated overnight at 37 °C with calcium ionophore A23187 (Sigma-Aldrich, St. Louis) at a final concentration of 10 μM. Following incubation, RBCs were gently collected from the flask, and cell debris was removed via a series of differential centrifugation steps: 600*g* for 20 min, 1600*g* for 15 min, 3260*g* for 15 min, and 10,000*g* for 30 min, all at 4 °C. At each step, the pellet was discarded and the supernatant was transferred to a new tube and filtered through 0.45 μm nylon syringe filters. EVs were then concentrated by ultracentrifugation at 100,000*g* for 70 min at 4 °C (Optima XPN-100, TY45 Ti rotor, Beckman Coulter). The resulting pellets were resuspended in cold PBS, layered over a 2 mL frozen 60% sucrose cushion, and centrifuged again at 100,000*g* for 16 h at 4 °C (Optima MAX-XP, MLS-50 rotor, Beckman Coulter), with the deceleration set to zero. The red EV-containing layer was collected, washed twice with cold PBS, and subjected to a final spin at 100,000*g* for 70 min at 4 °C (Optima MAX-XP, TLA-55 rotor, Beckman Coulter). The purified EVs were finally resuspended in 1 mL of cold PBS.

### SDS PAGE and Western Blot

For SDS-PAGE and Western blotting, samples were denatured by boiling for 5 min at 95 °C in reducing SDS sample buffer (80 mM Tris pH 6.8, 2% SDS, 7.5% glycerol, 0.01% bromophenol blue) supplemented with 2% 2-mercaptoethanol. Proteins were then separated on 10% acrylamide/bis­(acrylamide) SDS-PAGE gels. Densitometric analysis was performed using Image Lab software (Bio-Rad). For CTX quantification, gels were imaged postelectrophoresis using a Syngene G:BOX Chemi XX9 system, with a 2 min exposure at 667 nm. Following electrophoresis, proteins were transferred onto activated poly­(vinylidene difluoride) (PVDF) membranes for 1 h and then blocked with 5% (w/v) fat-free dried milk in PBS containing 0.05% Tween-20 (PBS-T) for 1 h at 37 °C. Membranes were incubated overnight at 4 °C with primary antibodies diluted 1:1000 in PBS-T containing 1% (w/v) fat-free dried milk. The first membrane was incubated with mouse anti-Band 3 (Santa Cruz Biotechnology, sc-133190) and rabbit anti-ApoA1 (Sigma-Aldrich, JS-251S1), while the second was incubated with mouse anti-ApoB100 (Santa Cruz Biotechnology, sc-393636). After incubation, the membranes were washed three times for 10 min with PBS-T and then incubated for 1 h at room temperature with horseradish peroxidase (HRP)-conjugated secondary antibodies (rabbit antimouse and goat antirabbit, Zymed) diluted 1:3000. Following three additional washes, chemiluminescence signals were acquired using Bio-Rad Clarity Western ECL on a G:BOX Chemi XT Imaging System (Syngene).

### BCA Assay

Total protein concentrations of REVs and LPs samples were determined with Pierce BCA Protein Assay Kit (ThermoFisher, Rockford), following the manufacturer’s instructions.

### Dynamic Light Scattering

Dynamic Light Scattering (DLS) measures the fluctuations in the intensity of light scattered by particles as they undergo Brownian motion. The scattered light intensity fluctuates over time due to the random motion of particles. Smaller particles move more rapidly than larger particles, causing faster fluctuations in the scattered light intensity. The relationship between the speed of Brownian motion and particle size is governed by the Stokes–Einstein eq (eq 1)­

1 $$D=\frac{K_{B}T}{6\pi \eta R}$$

where:

- *D* is the diffusion coefficient.
- *K*_B_ is the Boltzmann constant.
- *T* is the absolute temperature.
- η is the viscosity of the solvent.
- *R* is the particle hydrodynamic radius.

The size of the particles was inferred by calculating the diffusion coefficient from the fluctuation data. Additionally, measurements of ζ-potential were performed. All analyses were conducted using the Zetasizer Ultra (Malvern Panalytical, Malvern, U.K.). For size measurements, samples were diluted in PBS to a final concentration between 1–10 nM. One mL volume of the diluted sample was transferred into a DLS cuvette for measurement. Prior to each measurement, a 120-s equilibration step was applied to ensure sample homogeneity and temperature stability. After the acquisition, the correlogram and count rate were evaluated to confirm appropriate sample concentration. Data analysis was conducted considering intensity, volume, and number distributions. The polydispersity index (PDI) was also assessed to determine the width of the size distribution. For ζ-potential measurements, samples were diluted 1:10 in PBS. Before use, the ζ-potential cuvette was thoroughly rinsed with water. Then, 80 μL of the diluted sample was loaded into the cuvette, followed by three consecutive measurements steps of 30 s each.

### REV Labeling

Isolated EVs were labeled using the Atto 633 *N*-hydroxysuccinimidyl (NHS) ester fluorescent probe, an active ester derivative of Atto 633 (Sigma-Aldrich, St. Louis). The NHS ester reacts efficiently with compounds containing primary amines, forming a stable amide bond with membrane proteins on the EV surface. The dye was first dissolved in anhydrous *N*,*N*-Dimethylformamide (DMF) to a final concentration of 0.014 M. This stock solution was then diluted 1:1000 in PBS, and 24 μL of the diluted dye was added to an Eppendorf tube containing 1 mL of REVs. The final mixture was incubated overnight at room temperature on a shaker in the dark to ensure efficient labeling. Labeled EVs were subsequently stored at 4 °C until further use. To characterize the labeled samples, fluorescence spectra were acquired to determine the optimal detection range for fluorescence correlation spectroscopy (FCS). FCS measurements were then carried out to evaluate the ratio of free fluorophore in solution and to assess the hydrodynamic diameters of the labeled EVs.

### LP Labeling

1,2-dipalmitoyl-*sn*-glycero-3-phosphoethanolamine (DPPE) labeled with Atto 488 (Sigma-Aldrich, St. Louis), was resuspended in a chloroform/methanol mixture (8:2 ratio) to a final concentration of 0.001 M and stored at −20 °C. To prepare labeled lipoproteins (LPs), 1 μL of the fluorophore solution was mixed with 989 μL of PBS and 10 μL of LPs. This procedure was repeated for each lipoprotein type (HDL, LDL, and VLDL, all purchased from MyBioSource Inc., San Diego, CA). The resulting mixtures were incubated overnight at room temperature on a shaker in the dark to ensure efficient labeling. To characterize the labeled samples, fluorescent spectra were collected to determine the optimal detection range for fluorescence correlation spectroscopy (FCS). FCS measurements were performed to assess the ratio of free fluorophore in the solution and to determine the hydrodynamic diameters of the labeled LPs.

### Spectrofluorometry

The fluorescence spectra of labeled REVs and LPs were acquired using an FS5 spectrofluorometer (Edinburgh Instruments Ltd., Livingston, U.K.) to optimize the settings for FCS and FCCS measurements. Various standards of free fluorophores were prepared at concentrations of 0, 50, 100, 200, 400, and 800 nM for each fluorophore in order to build a calibration curve. Following this, samples were analyzed using the same settings to determine their concentrations. The experiment involved acquiring emission scans of both the standards and the samples. The excitation wavelengths were carefully set for each sample type to optimize fluorescence detection. For the REV sample and Atto NHS 633 standards, the excitation wavelength was set at 633 nm. For the lipoprotein samples and Atto DPPE 488 standards, the excitation wavelength was set at 488 nm.

### Sample Preparation

One hundred μL of each LP were mixed separately with 50 μL of red blood cell-derived extracellular vesicles (REVs). The mixture was incubated for 24 h at room temperature on a rotary mixer. Concentrations and ratios of REVs to LPs were chosen to best mimic physiological conditions, and specific particle concentrations for each technique are given in the related sections and in the main text. It is estimated that physiological concentrations of EVs, HDL, LDL, and VLDL are approximately 10^11^, 10^14^, 10^13^, and 10^12^ particles per milliliter, respectively, though these values can vary considerably due to factors such as circadian rhythms and pathophysiological conditions. The first measurements were conducted (t1, see Figure 1 in the main text). Subsequently, 25 μL of depleted human plasma was added to the mixture to achieve a final protein concentration of 3 μg/mL, followed by an additional 24 h of incubation and further measurements (t2). These analyses were designed to demonstrate the interaction between LPs and REVs in two different environments (PBS for t1 and plasma for t2, as shown in Figure 1 of the main text).

### dSTORM-DBSCAN SRM

Labeled REVs (Atto-647) and LPs (Atto-488) were used for this analysis, with final concentrations of 10^11^ EVs/mL for EVs, 5 × 10^12^ LPs/mL for LDL, and 5 × 10^11^ LPs/mL for VLDL. One hundred microliters (μL) of each, under both T1 and T2 conditions, were added to a freshly plasma-treated 18-well μ-Slide (Ibidi, Germany) and incubated for 1 h. After incubation, the supernatant was aspirated and replaced with a blocking buffer (1% BSA in PBS). Prior to imaging, the blocking solution was removed and replaced with 250 μL of freshly prepared standard STORM blinking buffer. For each sample, the blinking buffer was prepared freshly from stock solutions before imaging. The blinking buffer consisted of 10 mM MEA (Apollo Scientific Ltd.), 80 mgmL^–1^ glucose (Sigma–Aldrich, Germany), 9% glycerin (Sigma–Aldrich, Germany), 2 μgmL^–1^ catalase (Sigma-Aldrich, Germany), 100 μgmL^–1^ glucose oxidase (Sigma-Aldrich, Germany), 0.4 mM Tris­(2-carboxyethyl)­phosphine hydrochloride (Sigma-Aldrich, Germany), 2.5 mM KCl (Sigma–Aldrich, Germany), 2 mM Tris (Sigma–Aldrich, Germany) pH 7.5. The sample was covered with a glass coverslip to avoid the diffusion of oxygen inside. Samples were imaged using a Nikon Ti2 Eclipse inverted microscope system equipped with an ANDOR iXon DU897 EM-CCD camera (16 × 16 μm^2^ pixel size) and an SR Apochromat TIRF 100× 1.49 N.A. oil immersion objective. The sample was illuminated in total internal reflection mode, with the laser angle of incidence monitored by a Photometrics Dyno CCD camera at the back focal plane of the objective. Imaging was performed sequentially using 647 nm (125 mW) and 488 nm (80 mW) laser lines, with the laser power set to 40% at 2× magnification. A total of 10,000 images were recorded with an exposure time of 30 ms. The *XY* coordinates of each fluorescent event across the stack of 10,000 images were determined using the ThunderSTORM ImageJ plugin run using the default parameter settings. A Density-Based Spatial Clustering of Applications with Noise (DBSCAN) custom python script was then used to identify clusters corresponding to REVs or LPs from the SRM localization data sets and to evaluate REV-LP colocalization. This algorithm classifies points based on their local spatial density, allowing the detection of clusters. DBSCAN requires two user-defined parameters to proper work: epsilon (ε) and min_samples. ε defines the radius within which neighboring localizations are considered part of the same spatial neighborhood. Min_samples defines the minimum number of localizations required within ε for a point to be classified as part of a dense region (a “core point”). Localization points that meet these criteria are grouped into clusters, while points that do not reach the minimum density threshold are classified as noise. In our measurements, the parameters ε and min_samples were optimized empirically based on point-density distributions obtained from SRM data sets, from the REV and LP size, and from stoichiometric calculations performed starting from REV, LP and fluorescent probe concentrations. The ε and Min_sample values for each particle type are reported in Table 1.

**Table 1:** ε and Min_sample Values Used for DBSCAN

	REVs	HDL	LDL	VLDL
ε	60	4	8	15
Min_samples	50	2	2	40

For each SRM data set, DBSCAN was applied independently to the localization data sets of the green and red fluorescent channels, corresponding respectively to LPs and REVs. Finally, for each cluster identified as a REV or an LP, a convex hull was computed to define its spatial boundaries. The convex hulls of REVs and LPs present within the same field of view were then analyzed to assess spatial overlap and to quantify the percentage of colocalization

### Fluorescence Correlation Spectroscopy

Fluorescence Correlation Spectroscopy (FCS) is a highly sensitive analytical technique that utilizes fluorescence microscopy to measure fluctuations in fluorescence intensity. These fluctuations are caused by fluorescent molecules moving in and out of a small, defined confocal volume, illuminated by the laser. FCS provides quantitative information about molecular dynamics, including diffusion coefficients, concentrations, and molecular sizes. The correlation function is used to analyze fluorescence fluctuations, and the mathematical representation is reported in eq 2

2 $$G\left(\tau \right)=\frac{\left\langle \delta F\left(t\right)\delta F\left(t+\tau \right)\right\rangle }{{\left\langle F\left(t\right)\right\rangle }^{2}}$$

where:

- *G*(τ) is the correlation function.
- ⟨δ*F*(*t*)­δ*F*(*t* + τ)⟩ is the autocorrelation of fluorescence fluctuation.
- τ is the time lag.
- ⟨*F*(*t*)⟩ is the average fluorescence intensity over time.

One hundred microliters (μL) of samples were plated in an 8-well rectangular plate (Corning). The analysis was performed using the Zeiss NLO 880 (Group Zeiss, Oberkochen, GER), a fluorescence confocal microscope equipped with the following excitation lines: 405 nm, Argon laser (458, 488, 514 nm), DPSS 561 nm, 590 nm, and HeNe 633 nm. Measurements were carried out using GaASP and PMT detectors for single fluorescence molecule detection and dynamic characterization. The objective used was the C-APOCHROMAT (Group Zeiss, Oberkochen, GER), a water immersion objective with a magnification of 40× and a numerical aperture of 1.20. Prior to measurement, 10 μL of water were placed on the objective. Free fluorophores (ATTO488 and ATTO633) were analyzed to calibrate the instrument by adjusting the pinhole and determining the confocal volume. Depending on the sample concentration, the laser power was adjusted to achieve a count rate of approximately 200 kHz. The linearity between laser power and counts per molecule (CPM) was verified to establish the linear range of laser power before proceeding with the analysis. Following this setup, the FCS measurement was conducted with a measurement time of 10 s and 30 repetitions. To ensure statistical reliability, the series were repeated three times. After measurements, the resulting curves were fitted to appropriate models using QuikFit software to extract diffusion coefficients and hydrodynamic diameters.

### Fluorescence Cross-Correlation Spectroscopy

Fluorescence Cross-Correlation Spectroscopy (FCCS) is an advanced biophysical technique used to study molecular interactions, diffusion dynamics, and concentrations of biomolecules in solution or within cellular environments. It is an extension of FCS, measuring the simultaneous fluctuations of fluorescence signals from two spectrally distinct fluorophores in real-time. This technique provides quantitative information about molecular interactions, diffusion dynamics, and the number of interacting particles. The core of FCCS analysis is the cross-correlation function (CCF), which is mathematically expressed by eq 3

3 $$G_{\mathrm{xy}}\left(\tau \right)=\frac{\left\langle \delta F_{x}\left(\tau \right)\delta F_{y}\left(t+\tau \right)\right\rangle }{\langle F_{x}\left(\tau \right)\rangle \left\langle F_{y}\left(\tau \right)\right\rangle }$$

where:

- *G*_*xy*_(τ) is the cross-correlation function.
- δ*F*_*x*_(τ) and δ*F*_*y*_(τ) are the fluorescence intensity fluctuations over time for each fluorophore.
- τ is the time lag.
- *t* is the fixed time at which the measurement is starting to observe the quantity *F*_*y*_.
- ⟨·⟩ denotes the time average.

The cross-correlation can result in a positive correlation, indicating that the fluorescent species are moving together, or in a negative or zero correlation, suggesting no interactions or independent movement of the species. The analysis was performed with the same instrument and the same settings of FCS. The excitation of the two fluorophores was achieved using a HeNe 633 nm laser and an argon laser. The detector used was suitable for capturing the fluorescence emitted by both fluorophores. The procedure for the analysis was the same as FCS measurement. After measurements, the resulting curves were fitted to appropriate models, using QuikFit software to extract the cross-correlation data. The cross-correlation and the autocorrelation amplitudes were correct for free dye.^27^ The correction for the autocorrelation curve is done as indicated in eq 4

4 $$A_{0}=\frac{1}{N_{0}}=\frac{A\cdot I_{\mathrm{tot}}^{2}}{({I_{\mathrm{tot}}-I_{b})}^{2}}$$

where:

- *A*_0_ is the corrected FCS amplitude
- *N*_0_ is the corrected *N*
- *A* is the measured amplitude (before correction)
- *I*_tot_ is the total intensity
- *I*_b_ is the averaged background intensity

The correction of the cross-correlation curve is plot as described in eq 5

5 $${\mathrm{Acc}}_{0}=\frac{\mathrm{Acc}\cdot I_{\mathrm{tot},\mathrm{green}}\cdot I_{\mathrm{tot},\mathrm{red}}}{\left(I_{\mathrm{tot},\mathrm{green}}-I_{b,\mathrm{green}}\right)\cdot \left(I_{\mathrm{tot},\mathrm{red}}-I_{b,\mathrm{red}}\right)}$$

where:

- Acc_0_ is the corrected cross-correlation amplitude
- Acc is the measured cross-correlation amplitude before correction
- *I*_tot,green_/*I*_tot,red_ are the total intensities in the green and red channels, respectively
- *I*_b,green_ is the averaged background signal in the green channel
- *I*_b,red_ is the averaged background signal in the red channel

The fractions of interacting LPs and REVs have been calculated after the correction as described in eqs 6 and 7, respectively

6 $$\rho \mathrm{LPs}=\frac{\mathrm{Ac}c_{0}}{A_{0}\mathrm{REVs}}$$

7 $$\rho \mathrm{REVs}=\frac{\mathrm{Ac}c_{0}}{A_{0}\mathrm{LPs}}$$

Where:

- ρLPs is the fraction of interacting LPs
- ρREVs is the fraction of interacting REVs
- *A*_0_REVs is the corrected FCS amplitude of REVs
- *A*_0_LPs is the corrected FCS amplitude of LPs

### Flow Cytometry Bead Functionalization and Measurements

MagnaBind Carboxyl Derivatized Beads (Cat. No. 21353) were functionalized in two steps. First, DBCO-NH_2_ was covalently coupled to the beads by activating carboxylic groups with EDC. The beads were then incubated with MSP-N_3_. After each step, the magnetic beads were washed with PBS (pH 7.4) using a magnet support. For analysis, MSP-beads were incubated with 0.1 mL of the sample overnight at 10 °C and 800 rpm. Following incubation, three washing steps were performed on the magnetic support using Assay Buffer (PBS, 0.05% BSA). The beads were then resuspended in 0.5 mL of Assay Buffer and analyzed with a flow cytometer (CytoFLEX S). Two different fluorescent channels were used, for REVs (Atto647) bandpass filter: 660/10 nm, gain = 3000, and for LPs (Atto488) bandpass filter: 525/40 nm, gain = 3000. All the samples were analyzed at a medium flow rate of 30 μL min^–1^ for 60 s after mixing for 3 s.

### SiMoA Assay

SiMoA beads were conjugated with the Band 3 antibody following the Quanterix Homebrew kit protocol. For conjugation, 150 μL of carboxylated paramagnetic beads (2.8 × 10^9^ particles/ml) were washed three times with 300 μL of Bead Wash Buffer (Quanterix, phosphate buffer with detergent). After each wash, the beads were centrifuged and placed on a magnetic separator for 1 min to remove the supernatant. The beads were then washed three additional times with 300 μL of Bead Conjugation Buffer (Quanterix, 50 mM MES buffer, pH 6.2). Next, the beads were activated with 0.3 mg/mL EDC for 30 min at room temperature under continuous mixing or shaking. Following activation, 300 μL of an antibody solution—each antibody at a final concentration of 0.2 mg/mL—was added to the activated beads and incubated for 2 h at room temperature with mixing or shaking. After conjugation, the beads were washed twice with Bead Wash Buffer, then blocked with Bead Block Buffer (Quanterix, phosphate buffer with BSA) for 30 min at room temperature under continuous mixing or shaking. Following the blocking step, the beads were washed three times with Bead Diluent and stored at 4 °C until use. The three-step SiMoA assay was performed using anti-Band 3-conjugated beads (Santa Cruz, sc-133190), unlabeled EV–LP complex samples, and biotinylated detector antibodies—either anti-ApoA1 (Sigma-Aldrich, JS-251S1) or anti-ApoB100 (Santa Cruz, sc-393636). Conjugated beads were prepared at a final concentration of 2 × 10^7^ beads/ml in Bead Diluent. Then, 100 μL of each EV–LP sample was transferred to a 96-well microwell plate, followed by the addition of 25 μL of diluted beads. The plate was incubated at 25 °C for 30 min with shaking at 800 rpm on an orbital shaker before undergoing automated washing. Next, the beads were incubated with 100 μL of a biotinylated antibody mix (final concentration: 0.0003 mg/mL) for 10 min, followed by another automated wash. They were then incubated for 10 min with a 150 pM SBG solution (prepared in SBG Diluent, Quanterix), washed again, and inserted into the Quanterix SR-X instrument for analysis, where RGP was automatically added. Data were analyzed using SiMoA Reader Software version 1.10.

## Results and Discussion

### Biophysical and Biochemical Characterization of REVs and LPs

REVs and LPs were biophysically and biochemically characterized (Figure 2) using the BCA assay, Western blotting, and dynamic light scattering (DLS).^40,41^ REVs were isolated from blood samples following the protocol developed by Usman et al.,^42^ which employs calcium ionophore and Ca^2+^ to induce the vesiculation of red blood cells.^6,11^ The total protein content of REV samples, quantified using the BCA assay, was found to be 2.08 ± 0.12 μg/μL, while for human HDL, LDL, and VLDL samples the total protein concentration measured was 35.08 ± 2.31, 12.81 ± 2.14, and 4.17 ± 0.52 μg/μL, respectively. DLS, a widely applied technique for nanoparticle analysis, was used to determine the size distribution, ζ-potential, and particle concentration of both REVs and LPs. For this analysis, size data were interpreted based on particle count rather than intensity to avoid the dominance of high-intensity peaks from larger particles, thus providing a more accurate representation of nanoparticle populations. Results revealed the size distributions for all particle types, with hydrodynamic diameters (HD) averaging 120 nm for REVs (Figure 2A, blue curve), 30 nm for VLDL (Figure 2A, purple curve), 15 nm for LDL (Figure 2A, green curve), and 8 nm for HDL (Figure 2A, red curve). Additionally, all particles exhibited negative ζ-potentials in PBS (Figure 2B). The particle concentrations of the stocks were 7.66 × 10^11^ particles/mL for REVs (Figure 2C, blue column), 4.23 × 10^17^ particles/mL for HDL (Figure 2C, red column), 5.38 × 10^15^ particles/mL for LDL (Figure 2C, green column), and 2.92 × 10^13^ particles/mL for VLDL (Figure 2C, purple column). These findings are consistent with those reported in the literature, supporting the validity of the measurements and methodologies used in this study.^43–45^ Western blotting was employed to detect specific protein markers on REVs and LPs, and to exclude the cross-contamination of the samples. Ten μg of proteins for each sample were loaded on a polyacrylamide gel, electrophoresed, and transferred onto a poly­(vinylidene fluoride) (PVDF) blotting membrane. Primary antibodies targeting typical proteins of REVs and lipoproteins were used, followed by HRP-conjugated secondary antibodies for detection on the blotting membrane. For nanoparticle characterization, Band-3 was used as a biomarker for REVs, ApoA1 for HDL, and ApoB100 for LDL and VLDL (Figure 2D). REVs showed only Band-3 signal, indicating no lipoprotein contamination. HDL exclusively contained ApoA1, while both LDL and VLDL samples displayed only ApoB100, confirming the purity of each sample and the absence of REVs or other lipoprotein contaminants. These findings confirm the presence of the respective nanoparticles in each sample and exclude cross-contamination.

**Figure 2:**
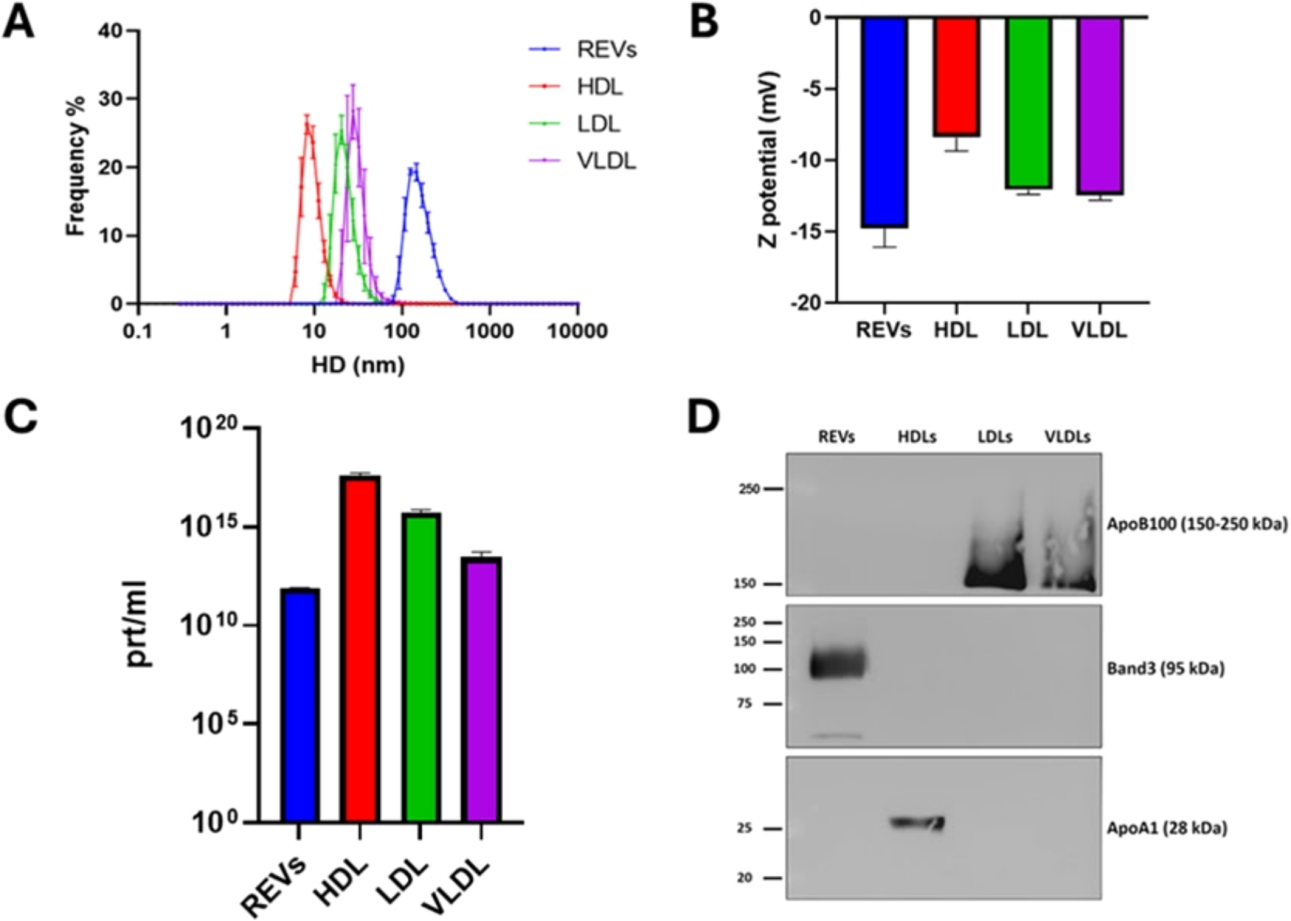
(A) Size distribution of REVs and LPs measured through DLS. (B) ζ-potential of REVs and LPs. All the particles exhibit a negative charge. (C) REV and LP concentrations determined by DLS. (D) Western Blot of specific biomarkers on REVs and LPs. REVs: Red Blood Cell EVs. HDL: High Density Lipoproteins. LDL: Low Density Lipoproteins. VLDL: Very Low Density Lipoproteins.

### REVs and LPs Fluorescent Labeling and Characterization

The fluorophores used in this work to label REVs and LPs for FCS, FCCS, SRM, and FACS measurements are Atto NHS 633 and Atto DPPE 488. The emission and excitation spectra of the free fluorophores were analyzed via spectrofluorometry to confirm no spectral overlap, ensuring accurate differentiation during subsequent analyses (SI, Figure S1A). REVs and LPs were labeled respectively with Atto NHS 633 and Atto DPPE 488, by following the protocols described in the SI Experimental Section. The characterization of the labeled materials is reported in SI, Figures S1 and S2.

### dSTORM-DBSCAN SRM Visualization of EV–LP Complexes

dSTORM SRM was employed to assess the presence of EV–LP complexes after the mixing of REVs with LPs, achieving single-particle resolution (Figure 3). In the following, the technical details of the methodology introduced in Figure 1 are described. The indicated testing conditions have been applied consistently in all subsequent experiments unless stated otherwise.

**Figure 3:**
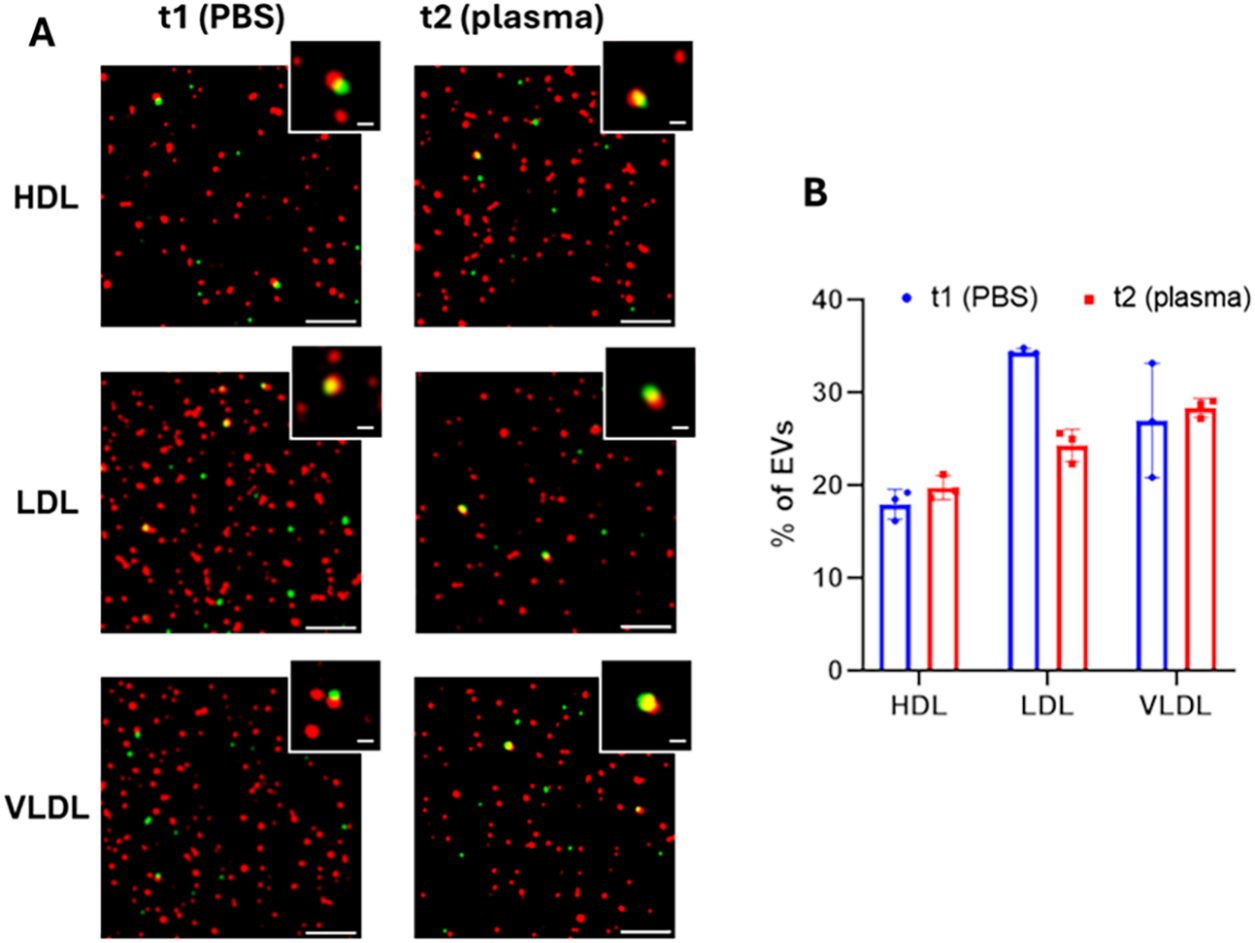
(A) Representative dSTORM images showing EVs in red, LPs in green and colocalized events in yellow. Scale bar: 2 μm for images, 300 nm for insets. (B) Percentage of EVs interacting with at least one LP, determined through DBSCAN. The *Y*-axis represents the percentage of colocalization events relative to the total number of EVs detected. *n* = 3 independent experimental replicates. REVs: fluorescent Red Blood Cell-derived EVs. HDL: fluorescent HDL. LDL: fluorescent LDL. VLDL: fluorescent VLDL.

100 μL of each type of LPs (corresponding to 1.04 × 10^13^ HDL, 4.60 × 10^12^ LDL, and 6.47 × 10^11^ VLDL) were mixed with 50 μL of REVs (corresponding to 3.83 × 10^10^ REVs) and incubated under mixing for 24 h at room temperature in sterile PBS. Such concentrations were chosen to mimic ENP physiological ratios at best. Indeed, it is estimated that physiological average concentrations of EVs, HDL, LDL, and VLDL are respectively 10^11^, 10^14^, 10^13^, and 10^12^ prt/ml, with an intrinsic variability due to circadian cycle and pathophysiological conditions.^46,47^ SRM measurements were then performed (t1). Subsequently, 25 μL of human plasma depleted of extracellular nanoparticles were added to the mixture to a final concentration of 3 μg/mL of proteins, followed by 24 h of incubation and additional SRM measurements (t2).^11^

Representative images of EV–LP binding for each sample are shown in Figure 3A. Colocalization of fluorophores was detected at both t1 and t2, indicating potential binding between EVs and LPs. The percentage of EV–LP complexes was calculated through a DBSCAN algorithm applied to the SRM data sets and plotted as a percentage of colocalization events over the total number of EVs detected (Figure 3B). The use of DBSCAN has several advantages compared to the simple superimposition of reconstructed SRM images and subsequent calculation of colocalizing particles, as it is less sensitive to errors introduced by image reconstruction and manipulation (e.g., sensitivity-to-contrast settings).^48^ Representative DBSCAN maps are reported in the Supporting Information (Figure S9).

As shown in Figure 3B, approximately 20% of the total REVs colocalized with HDL, while LDL and VLDL samples exhibited slightly higher interaction at around 30%. No significant difference in the colocalization rate was observed between t1 and t2 samples. These data serve as a first validation of our bottom-up approach to studying EV–LP binding, demonstrating that, under the tested conditions, REV-LP complexes form and can be detected.

### Determination of REV-LP Interaction Dynamics in Solution Using Fluorescence Cross-Correlation Spectroscopy

Despite visualizing the REV-LP interaction at the single particle level, SMR measurements cannot determine the nature or the dynamics of these interactions due to the nonspecific immobilization of particles on a surface for their detection (see Supporting Information file): for this reason, we decided to analyze REV and LPs interaction in more physiological conditions. We implemented FCCS analysis (Figure 4), an advanced *in situ* analytical technique (principle sketched in Figure 4A) used to study dynamic interactions between fluorescent molecular species in solution at the ensemble level. Fluorescent REVs and LPs were mixed in physiological ratios, as stated above, and measured following the experimental workflow shown in Figure 1. Data analysis of correlograms and cross-correlograms (detailed in the SI, and represented in Figure S4) provided, for each sample, the fraction of interacting NPs. As shown in Figure 4B, the fraction of interacting LPs is small (between 0.01 and 0.02) for every LP considered. This data could be explained by the stoichiometric excess of LPs present in each preparation compared to REVs. Furthermore, no significant differences were detected between t1 and t2, indicating that plasma addition to the preparation did not impact the total number of LPs interacting with REVs. An analogous behavior between t1 and t2 has been observed with the interaction of REV with LDL and VLDL, with a tendency to increase that is not statistically significant. On the other hand, in the case of HDL, the fraction of interacting REVs showed significant increase between t1 and t2 (ranging from 0.45 to 1, black bars in Figure 4C), despite no differences in the fraction of interacting LPs at the same time points (black bars in Figure 4B). This suggests that the addition of plasma leads to a redistribution of HDL rather than the recruitment of new HDLs onto the REV surface. This behavior indicates that plasma proteins may perturb the preformed equilibrium between HDL and REVs, likely promoting partial displacement of HDL already adsorbed on the REV surface, followed by their readsorption onto other REVs.

**Figure 4:**
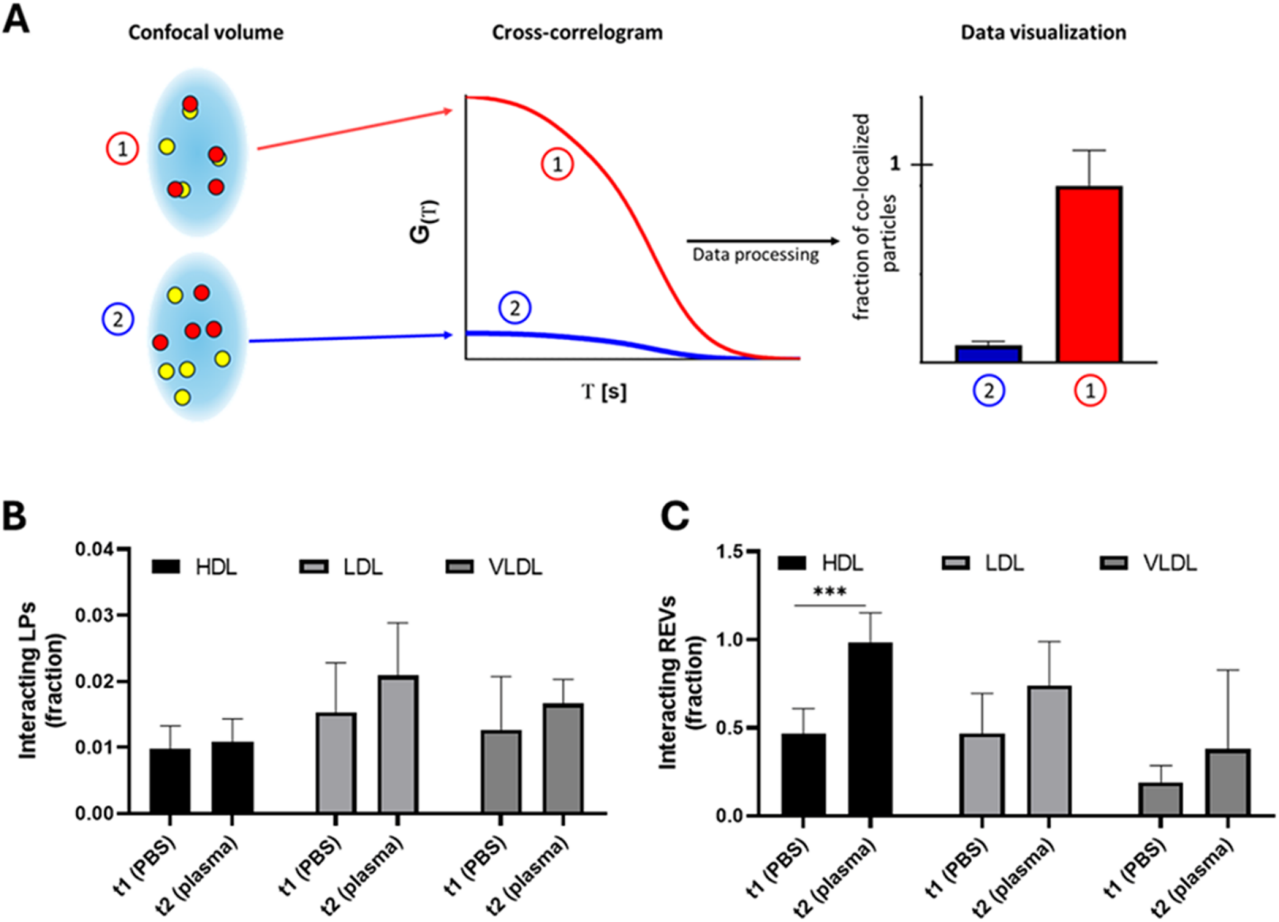
(A) Schematic representation of the FCCS principle. Fluorescently labeled particles (red and yellow) diffuse through a detection confocal volume, and their fluorescence intensity is recorded over time. Simultaneous detection of red and yellow fluorescence signals indicates particle–particle interaction. Cross-correlation analysis reveals the extent of this interaction between the two species, with a higher bar representing stronger correlation and higher interaction. (B) Fraction of interacting LPs (HDL in black, LDL in light gray, and VLDL in dark gray) at t1 (in PBS) and at t2 (in plasma). *n* = 3 independent experimental replicates. (C) Fraction of interacting REVs (HDL in black, LDL in light gray, and VLDL in dark gray) at t1 (in PBS) and at t2 (in plasma). *n* = 3 independent experimental replicates. Statistical significance of each group was tested using paired student’s *t* test. *** = *p* < 0.002. Nonsignificant differences are not indicated. Legend: REVs: fluorescent Red Blood Cell-derived EVs. HDL: fluorescent HDL. LDL: fluorescent LDL. VLDL: fluorescent VLDL.

We speculate that this phenomenon occurs specifically with HDL due to their small size, which is comparable to that of certain plasma proteins, and protein aggregates. This similarity may facilitate competition between plasma components for binding sites on the REV surface, thereby displacing weakly adsorbed HDL, which are subsequently redistributed among REVs in the solution.^49^ Finally, when evaluating the data obtained at t2, which more closely mimics physiological conditions, we observed different fractions of interacting REVs depending on the type of interacting LP. Specifically, HDL exhibited the highest fraction of interacting REVs (0.99 ± 0.17, black bar in Figure 4C), LDL showed an intermediate value (0.74 ± 0.25, light gray bar in Figure 4C), and VLDL had the lowest (0.38 ± 0.45, dark gray bar in Figure 4C). Based on these data, we calculated the average number of interacting LPs per REV for each LP type, estimating that approximately 30 HDL particles interact per REV, compared to 10 for LDL and only 1 for VLDL. Details on the calculation process are reported in the Supporting Information.

We identified two distinct correlations in these data: (i) a direct correlation with the number of LPs in the preparation, which is highest for HDL, intermediate for LDL, and lowest for VLDL; and (ii) an inverse correlation between LP size and the ρ of interacting REVs, suggesting that larger LPs, such as LDL and VLDL, may have limited binding due to steric hindrance.

FCCS data indicate that, in PBS and in physiological-like conditions, REVs (and possibly every EV) statistically interact with LPs of different types, confirming SMR results, validating our bottom-up model, and supporting the literature.^5,50,51^ Furthermore, FCCS, contrary to SMR, measures the interaction in solution avoiding surface immobilization, enabling the discovery of dynamic aspects of EV–LP complex formation. Therefore, we performed dose–response experiments to evaluate the apparent affinity constant for REV-LP binding (Figure 5). Apparent affinity constants are values that qualitatively describe the dynamic of binding between two components.^52–54^ Thus, comparing these values for each LP we can estimate which LP is more prone to interact with REVs in the tested conditions. 100 μL of seven different LP concentrations were tested, each mixed with a fixed amount of REVs and plasma, following the protocol described in the experimental section. For HDL, the concentrations used ranged from 3.03 × 10^12^ to 2.81 × 10^15^ prt/ml. For LDL, concentrations ranged from 1.30 × 10^12^ to 1.24 × 10^15^ prt/ml, while VLDL concentrations ranged from 2.21 × 10^11^ to 1.74 × 10^14^ prt/ml. FCCS results are expressed as fraction (ρ) of interacting REVs as a function of the LP concentration. Dose–response curves at t2 are reported in Figure 5A (red for HDL, green for LDL, and purple for VLDL, dose–response curves at t1 are reported in SI, Figure S5A).

**Figure 5:**
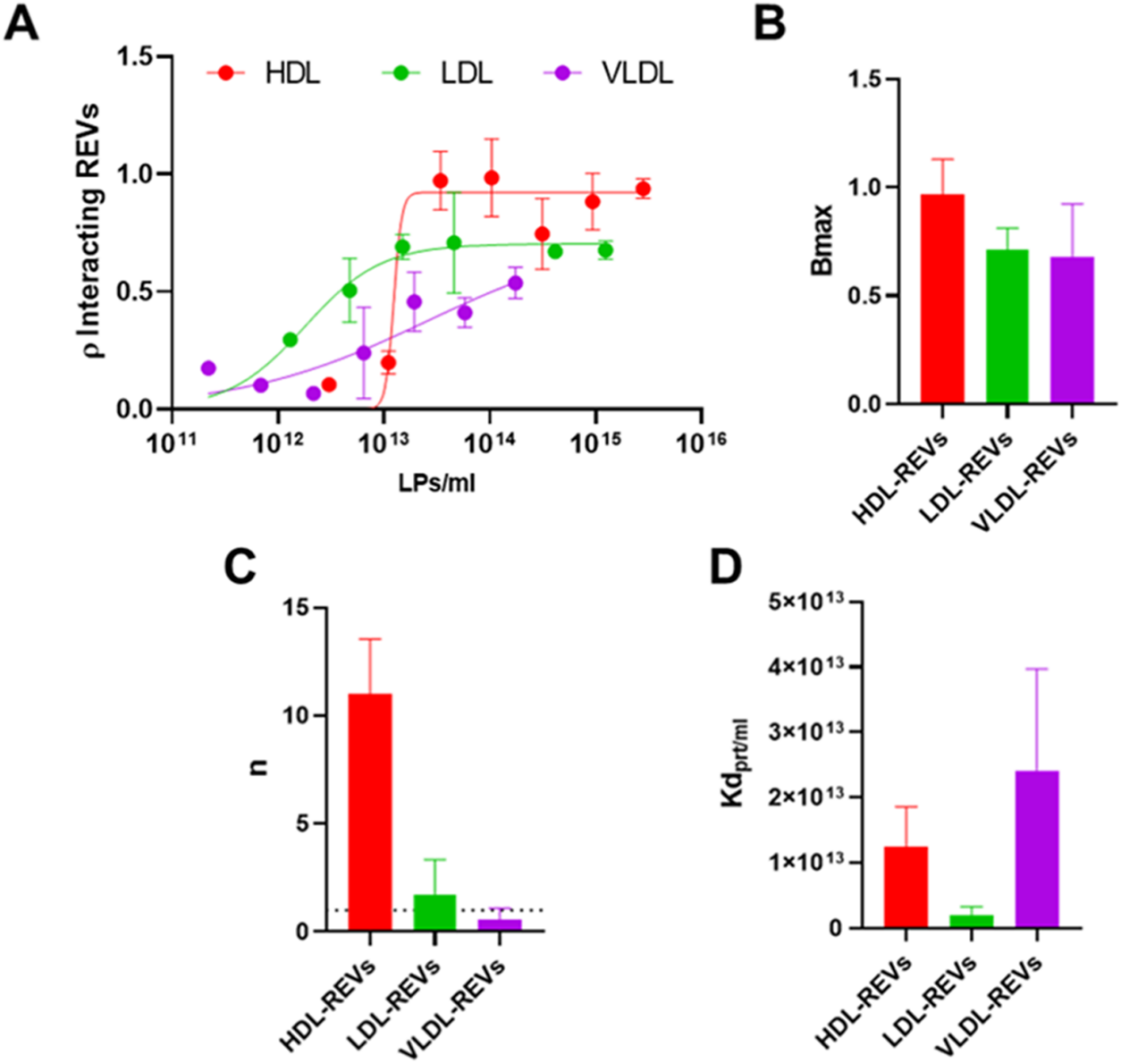
(A) Dose–response curve of EVs-LPs binding fitted with the Hill model (red for HDL, green for LDL, and purple for VLDL) at t2. *n* = 3 independent experimental replicates. (B) Graphical representation of Bmax extrapolated from the fitting for each EV–LP binding (red for HDL, green for LDL, and purple for VLDL). (C) Graphical representation of n extrapolated from the fitting for each EV–LP binding (red for HDL, green for LDL, and purple for VLDL), the dotted line represents *n* coefficient = 1. (D) Graphical representation of *K*_d_ extrapolated from the fitting for each EV–LP binding (red for HDL, green for LDL, and purple for VLDL). Legend: REV: fluorescent Red Blood Cell-derived EVs HDL: fluorescent High Density Lipoproteins. LDL: fluorescent Low Density Lipoproteins. VLDL: fluorescent Very Low Density Lipoproteins.

Data were fitted by applying the Hill model, which is used to describe cooperative binding or interaction phenomena and it has been already applied for describing BC formation dynamics on synthetic nanoparticles.^50,51^ The model assumes the binding of a ligand to one site can influence the binding of additional ligands to nearby sites, leading to either positive or negative cooperation.^55^

The Hill model is described by the eq 8

8 $$\rho =\frac{{\left[L\right]}^{n}}{K_{d}+{\left[L\right]}^{n}}$$

where:

- ρ: is the fraction of occupied binding sites,
- [L]: is the concentration of the ligand,
- *K*_d_: is the apparent dissociation constant,
- *n*: is the Hill coefficient, which indicates the degree of cooperativity. Specifically, if *n* *=* 1: no cooperativity, the binding follows the classical Langmuir behavior (independent binding). *n* *>* 1: positive cooperativity, meaning that the binding of one ligand increases the affinity of other ligands for nearby sites. *n* *<* 1: negative cooperativity, where the binding of one ligand decreases the affinity of other ligands for nearby sites.

It is important to note that, considering the complexity of EV–LP association, the Hill model might be a simplified way to interpret such interaction, and may not perfectly represent this system. Still, it provides information to qualitatively compare the different EV–LP complexes considered in this study.^50,51^

From the fitting curves, we extrapolated for each REV-LP complex three key parameters: (i) Bmax, which represents the maximum binding capacity of the system, corresponding to the binding signal reached under saturation conditions. In our system, Bmax would reflect the maximum binding capacity of the REV surface for LPs. (ii) n: the Hill coefficient described in the previous paragraph, here used as a fitting parameter, and (iii) K_d_, the apparent dissociation constant.

As depicted in Figure 5B, the binding between HDL and REVs shows a higher Bmax compared to LDL and VLDL, which exhibit similar values. This finding supports the hypothesis of steric limitations in the binding of LDL and VLDL with REVs. Indeed, although all LPs in this study likely have access to the same binding surface on REVs, the larger size of LDL and VLDL may hamper access to some of these sites due to steric hindrance, thus reducing the effective number of available binding sites. Interestingly, the analysis of the Hill coefficient revealed distinct binding mechanisms for each LP (Figure 5C). The HDL-REV interaction has an *n* > 1, indicative of cooperative binding. This is consistent with the similar size of HDL to plasma proteins, which typically adsorb onto surfaces forming a biomolecular corona (BC) that can promote the aggregation of additional proteins,^56,57^ as recently demonstrated.^11^ In contrast, the LDL-REV interaction displays an *n* ≈ 1, suggesting a Langmuir-like binding, which reflects a reversible, noncooperative, and saturable interaction.^53^ Finally, the VLDL-REV interaction, characterized by *n* < 1, indicates negative cooperation. This might be related to the larger size of VLDL, similar to REV one, which leads to steric and limitations in the binding, possibly due to analogous surface curvatures.^58^ A comparison of apparent *K*_d_ values (Figure 5D) reveals that LDL exhibits the lowest *K*_d_, and, in turn, the highest affinity for REVs, while HDL and VLDL display affinities approximately an order of magnitude lower. This observation is particularly intriguing, especially considering previous studies identifying LDL-EV binding as the most biologically relevant in the physiological environment.^5^ Indeed, LDL-EV aggregation is one of the few biogenic nanoparticle interactions with established biological significance. Busatto et al. demonstrated that cancer-derived EVs form aggregates with LDL, facilitating their passage across biological barriers, such as the blood-brain barrier.^26^

The data provided indicate that FCCS is a valuable tool for studying the ensemble dynamic binding between REVs and LPs, due to the measure being performed *in situ*, directly in solution, and without washing steps that could perturb the equilibrium established between the nanoparticles. Unfortunately, FCCS is not able to describe the nature of interaction occurring between EVs and LPs (adsorption, kissing/fusion, etc.). Plus, it requires fluorescently labeled particles, limiting its application onto real samples without performing time-consuming and sample-tailored optimization of staining procedures. Therefore, to overcome these limitations and complement FCCS data, *ad-hoc* novel FC method and SiMoA assays were put in place.

### EV–LP Binding Stability and Impact on EV Membrane Accessibility

Bead-based flow cytometry (FC) is a widely used method for evaluating EVs and their marker expression. Compared to FCCS measurements, FC analysis requires multiple washing steps to minimize nonspecific signal detection. Washing, with a physiological solution, challenges the LP-EV binding stability. Therefore, in our experimental flow, FC is used to evaluate the stability of the formed EV–LP complexes, complementing FCCS dynamic data (Figure 6).

**Figure 6:**
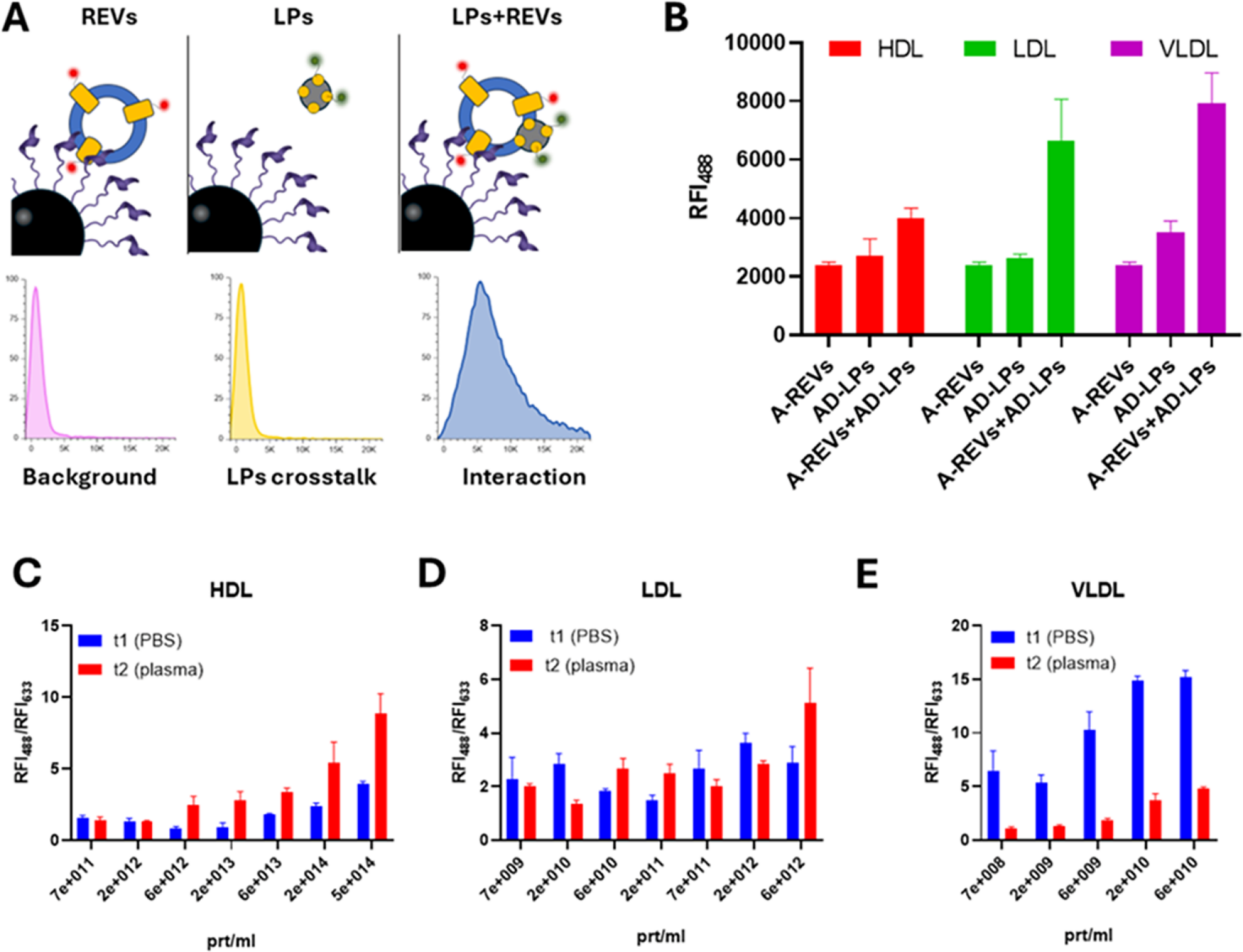
(A) Sketched representation of the FC measurement, with MSP-beads capturing EV–LP complexes via EV binding. (B) Negative and positive controls for each LPs are shown. MSP-beads captured REVs with minimal signal detected in the LP channels. Minimal nonspecific binding was observed between MSP and LPs (in red HDL, in blue LDL, and in violet VLDL). Signal intensity notably increased when EVs and LPs were incubated together; in each case, REVs-LDL and REVs-VLDL complexes displayed higher signals compared to their controls. *n* = 3 independent experimental replicates. (C) Interaction of REVs and HDL at different HDL concentrations. *n* = 3. (D) Interaction of REVs and LDL at different LDL concentrations. *n* = 3 independent experimental replicates. (E) Interaction of REVs and VLDL at different VLDL concentrations. *n* = 3 independent experimental replicates. The blue bars represent measurements at t1 (PBS), while the red ones at t2 (plasma). The interaction is reported as the ratio between the relative fluorescence in the blue and the red channels (RFI488/RFI633). Legend: REV: fluorescent Red Blood Cell-derived EVs HDL: fluorescent High Density Lipoproteins. LDL: fluorescent Low Density Lipoproteins. VLDL: fluorescent Very Low Density Lipoproteins.

To capture EVs and EV–LP complexes, we utilized Membrane-Sensing Peptide (MSP)-functionalized beads (hereafter MSP-beads, see SI),^60^ as their use was previously validated for REV samples. MSP is specific and selective for highly curved lipid bilayers, binding to membrane defects generated by such curvature.^59^ Conversely, they exhibit minimal binding to other EV surface components, such as transmembrane proteins, as well as with lipid monolayers like those surrounding LPs.^60^ The rationale of the FC measurement is sketched in Figure 6A.

Before analyzing EV–LP complexes, we verified the crosstalk between MSP beads and LPs by mixing the beads with the highest LP concentration used in our measurements and collecting the fluorescent signal at 488 nm (indicative of labeled LPs). As shown in Figure 6B (“LPs” bars), this signal is comparable to that of REV samples (which are labeled in red and scarcely detectable on the 488 nm channel, “REVs” bars), indicating negligible crosstalk between LPs and MSP beads. A detectable signal in the LP channel was observed only in the presence of REVs and LPs, (Figure 6B, “REVs + LPs” bar) further confirming SRM and FCCS data on EV–LP binding.

Then, we performed dose–response experiments by mixing a fixed amount of REVs with varying LPs concentrations, ensuring coverage of the physiological EV/LP ratios.

Interestingly, the intensity of the REV fluorescence signal (633 nm, red channel) as a function of LP concentration (SI, Figure S6)—which was expected to remain constant—gradually decreased with increasing LP concentrations. This trend suggests that at higher LP/REV ratios, LPs interact more extensively with the EV surface, reducing the available free surface area of EVs. As a result, fewer binding sites remain accessible for MSP to bind to the EV lipid bilayer, leading to reduced capture and a lower REV fluorescence signal. This observation is consistent with MSP’s known binding mechanism, and again supports the hypothesis that LPs interact with the EV surface. Remarkably, the LP concentration at which MSP capture becomes less effective correlates with LP size: the smaller the LP, the higher the concentration required to affect MSP binding. This suggests the involvement of EV surface saturation and steric hindrance mechanisms.^11^

Given its dependency on the number of captured REVs, the LP fluorescence signal (488 nm) was normalized to the fluorescence signal in the REV channel (633 nm) and expressed as the ratio of the two Relative Fluorescence Intensities (RFI_488_/RFI_633_, Figure 6C–E).

HDL showed increased interaction with EVs in t2 compared to t1, indicating even small amounts of plasma proteins could positively affect REV-HDL binding. Moreover, in both conditions, we observed a positive dose–response trend (Figure 6C). These data, although of a different nature, are in good agreement with FCCS results, which described a cooperative binding between HDL and EVs. Differently, LDL showed similar interactions between t1 and t2, suggesting a scarce effect of plasma proteins on the REV-LDL binding in the tested conditions. Moreover, a clear dose-dependent trend is missing (Figure 6D).

For VLDL, the signal is greatly reduced in t2 compared to t1, suggesting a detrimental effect of plasma proteins over the REV-VLDL adsorption. Moreover, in both t1 and t2, the interaction shows an initial positive dose–response trend but dramatically decreases upon reaching a VLDL/REV ratio of ∼10:1 (Figure 6E). Once again, FC data align well with FCCS results, which indicated a negative cooperative binding between VLDL and REVs. Indeed, while steric hindrance may limit the binding between VLDL and REVs above certain ratios, it could also restrict MSP access and binding to the EV membrane, ultimately leading to an overall reduction in fluorescence signals.

### Immunodetection of EV–LP Interaction

SiMoA technology is a digital platform for single molecule/particle detection based on immunoaffinity. Unlike FCCS, which requires directly labeled particles, and FC, which has lower sensitivity and requires a larger sample volume, SiMoA allows for the detection of unlabeled samples, achieving single-particle resolution through immunostaining.

To detect and study the binding of unlabeled REVs and LPs, we developed a custom SiMoA assay based on an immuno-hetero sandwich approach (Figure 7). This assay uses anti-Band 3 antibodies immobilized on beads to capture REVs and detecting antibodies against ApoA1 and ApoB100 to specifically identify HDL, and LDL or VLDL, respectively (concept sketched in Figure 7A).

**Figure 7:**
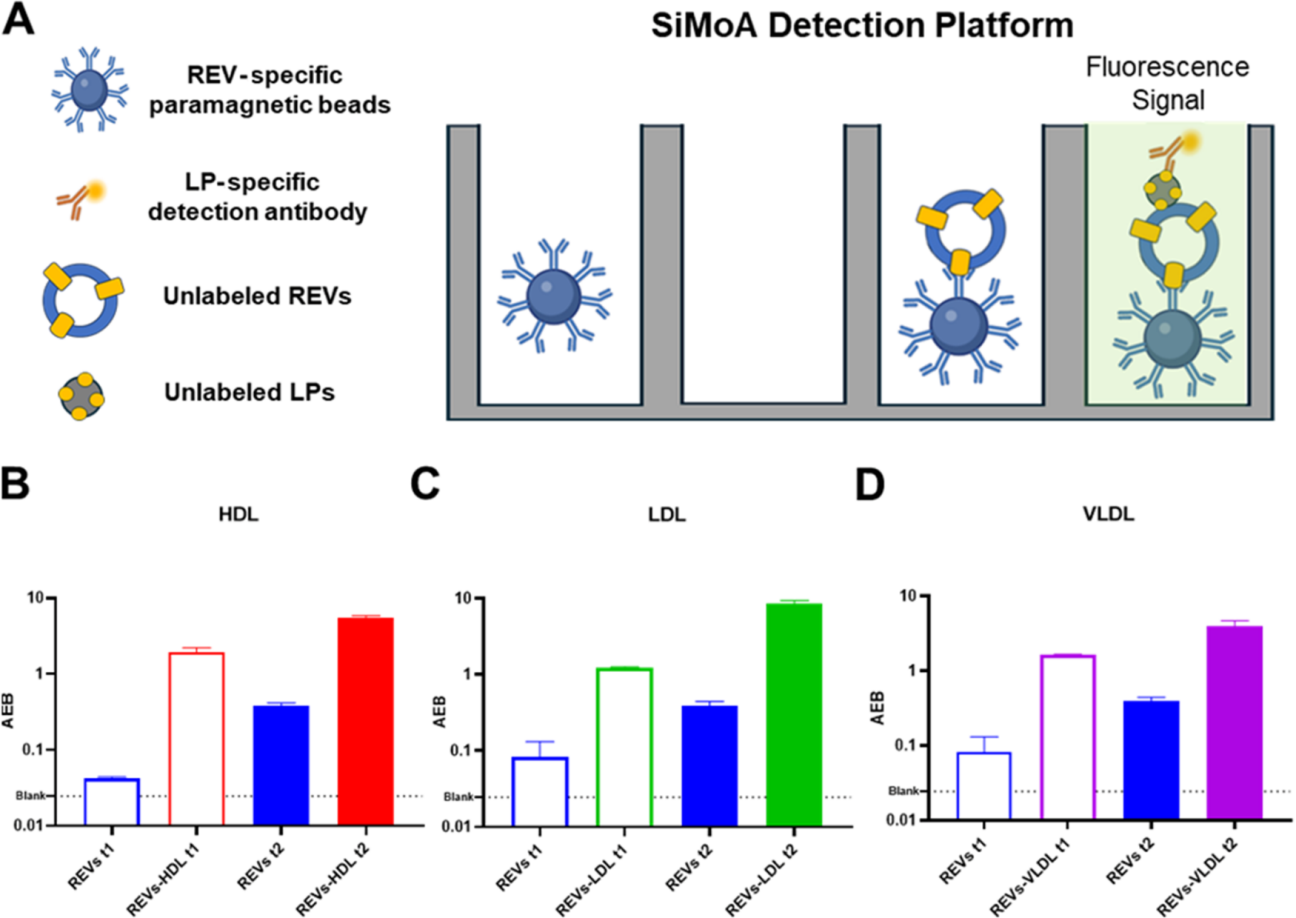
(A) Sketched representation of the proposed SiMoA assay to detect EV–LP binding. (B) Control tests to assess nonspecific signals for HDL. (C) Control tests to assess nonspecific signals for LDL. (D) Control tests to assess nonspecific signals for VLDL. for all the graphs. Data are expressed in Average Enzyme per Bead (AEB). *n* = 2 independent experimental replicates. Legend: REV: unlabeled Red Blood Cell-derived EVs HDL: unlabeled High Density Lipoproteins. LDL: unlabeled Low Density Lipoproteins. VLDL: unlabeled Very Low Density Lipoproteins.

Control experiments were conducted to investigate nonspecific signals. Anti-Band 3 beads were first incubated with anti-ApoA1 or anti-ApoB100 antibodies to set the background nonspecific noise (dotted line in Figure 7B–D) of the assay. Then pristine REVs at t1 and t2 were tested (REVs t1 and REVs t2 columns in Figure 7B–D). In REVs t1, ApoA1 and ApoB100 signals were negligible and close to background noise, as expected. In REVs t2, higher signals were detected for all tested LPs, suggesting the presence of a residual small amount of LPs in the plasma, regardless of the detection protocols. Among the residual LPs, HDL (containing ApoA1) seem to be the most represented. This is in line with HDL being very small ENPs, thus making their depletion through centrifugation and filtration less efficient.

When standard LPs were added to REVs t1 and t2, the signal increased approximately 10-fold in both conditions compared to the controls (Figure 7B–D), despite the incomplete depletion of LPs from plasma. This suggests that the residual LPs are limited and insufficient to saturate the REV surface.

Thus, all samples showed higher signals than controls in the same conditions, confirming the presence of interactions and validating this novel immunoaffinity assay for studying EV–LP complexes in physiological conditions and without using labeled materials.

We then conducted SiMoA dose–response experiments at t2 using the same stoichiometric ratios analyzed in FC that mimic physiological conditions, to determine the assay’s limit of detection (LOD) in the presence of plasma proteins (Figure 8). In all conditions tested, a dose-dependent response was observed (Figure 8A), indicating that the assay enables the detection of EV–LP complexes across a wide range of stoichiometric ratios. Notably, the dose–response curve obtained by SiMoA follows different trends compared to the ones obtained by FCCS, as this assay relies on immunoaffinity capture of the nanoparticles rather than direct fluorescence detection. From the dose–response curves, we also extrapolated the LOD for each LP-REV binding (Figure 8B), which is, for all the LPs, at least 1 order of magnitude below the physiological average concentration of LPs in plasma.

**Figure 8:**
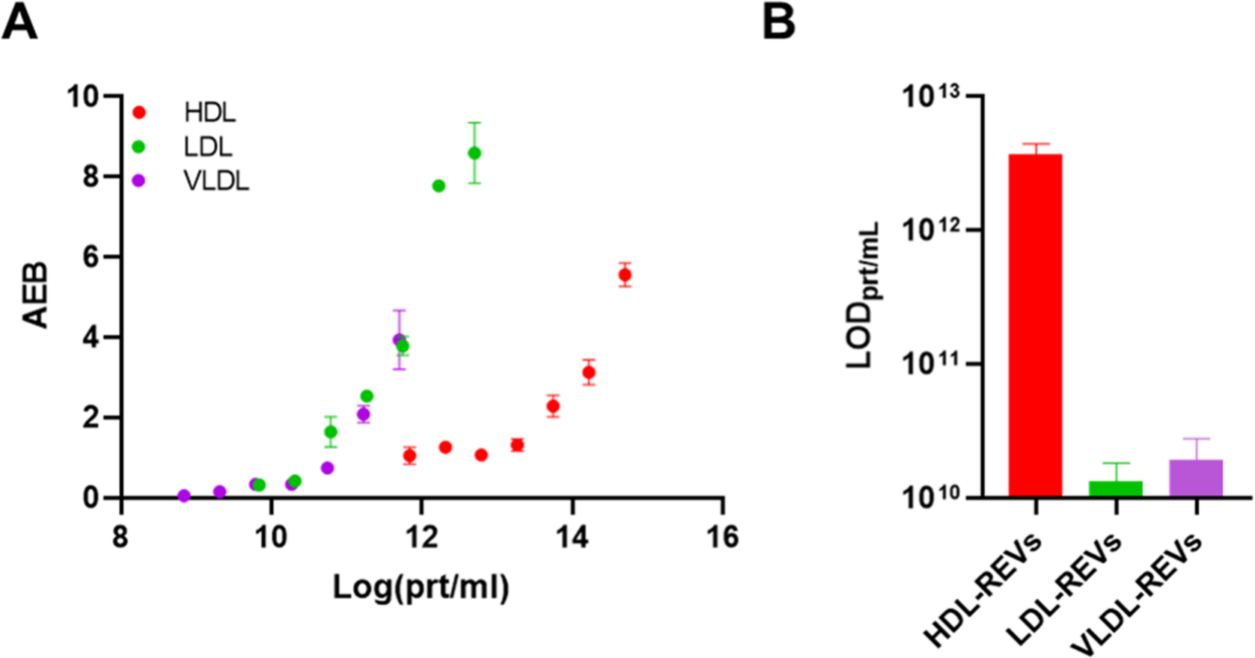
(A) Interaction of REVs and LPs at t2 (plasma) measured at different LP concentrations through immune heterosandwich SiMoA assay. Data are expressed in Average Enzyme per Bead (AEB). *n* = 2 independent experimental replicates. (B) LOD of each REV-LP interaction extrapolated from the curves shown in (A) Legend: REV: unlabeled Red Blood Cell-derived EVs HDL: unlabeled High Density Lipoproteins. LDL: unlabeled Low Density Lipoproteins. VLDL: unlabeled Very Low Density Lipoproteins.

Although the results obtained with *in situ* and *ex-situ* techniques often cannot be directly compared quantitatively, the consistency we observed among different techniques could suggest that EV–LP binding is not strongly affected by the methodological differences between FCCS and SiMoA. This, combined with the assay’s high sensitivity, makes it particularly valuable for future analytical applications, including its direct use on complex matrices and biological samples.

## Conclusion

Extracellular vesicles (EVs) possess an identity that extends beyond their molecular composition and cellular origin, encompassing mesoscale characteristics such as the biomolecular corona. These structural and functional attributes play a crucial role in defining EV interactions within biological environments. While the formation of biomolecular coronas is often regarded as an unintended side effect in synthetic nanoparticles, in EVs, this phenomenon appears to be an evolutionarily conserved property that significantly contributes to their biological function. In this study, we developed a bottom-up approach and a set of orthogonal analytical techniques to characterize the interactions between REVs and LPs. Our findings provide key insights into nature, stability, and potential implications of these interactions in physiological conditions. SRM first visualized EV–LP interaction down to the single particle level, allowing for a first determination of the entity of the binding. FCCS further revealed that REVs bind lipoproteins in solution, with affinities ranging from 10 nM to 1 μM, highlighting differential binding across LP classes, with up to 100% interaction in specific conditions. FC provided evidence that EV–LP binding is sufficiently stable to resist multiple washing steps, suggesting that lipoproteins may form a persistent corona around EVs. This association can affect recognition processes at the EV membrane, as indicated by the reduced binding capacity of membrane-sensitive peptide at high LP concentrations. On the contrary, the stable binding of LPs onto the EV surface could improve the recognition of EV–LP complexes by specific receptors, such as SR-BI or LDL-R, contributing to EV uptake and biodistribution into specific body districts. Finally, the integration of Single Molecule Array (SiMoA) technology with a novel immune-hetero sandwich approach enabled the measurement of EV–LP binding. This strategy preserves the native interaction states of EVs and lipoproteins, further strengthening FCCS and FC results and opening for the study providing a valuable tool for studying EV–LP interactomes in physiologically relevant conditions and complex biological samples. Taken together, our findings suggest that EV–LP binding is not limited to a specific LP class (e.g., LDL, as previously reported), it is rather a general phenomenon, shared, with different extents, by all LP classes. This implies that EVs in the bloodstream may be constantly associated with a certain number of lipoproteins, with possible impacts on EV surface identity and, therefore, function and biodistribution. Our study contributes to a deeper understanding of the extracellular nanoparticle interactome, revealing that EV-lipoprotein binding is shaped not only by the physicochemical properties of EVs themselves but also by the surrounding biological environment. However, given the context-dependent nature of these interactions, we cannot exclude that the results reported here are partly influenced by the specific experimental conditions applied, and that different outcomes might emerge upon varying some of these parameters. Nevertheless, the present work provides a solid analytical framework for deciphering such dependencies, which future investigations can benefit from. In fact, the analytical strategy developed here offers a versatile platform to broaden the EV–LP interaction landscape. Future studies could, for example: (i) probe sequential or competitive LP binding to EVs; (ii) dissect the specific plasma proteins that drive these interactions; (iii) explore the implications of EV–LP association for the in vivo functionality of these complexes; and (iv) support the rational design of EV-based or hybrid drug-delivery systems, ultimately advancing the clinical translation of extracellular vesicle technologies. Regarding this last point, the design of “decoy EVs” with selective lipoprotein affinity could be used to target and saturate phagocytic cells, thereby preventing the rapid clearance of therapeutic EVs from circulation.^61–63^ This could improve the delivery of EV-based therapeutics to hard-to-reach sites, overcoming key challenges such as unwanted accumulation in the liver, spleen, and kidneys.

## Supplementary Material



## Notes

Additional experimental details on REV and LP characterization, calculation of the number of interacting LPs per REV, determination of REV-LP interaction dynamics, and EV–LP binding stability, label-free immunodetection of EV–LP interaction, representative DBSCAN maps (PDF)
