# Supplementary material for: Orthogonal Investigation at Single-Particle and Ensemble Levels Uncovers Lipoprotein-Extracellular Vesicle Binding
Source: Anal Chem. 2026 Jan 8;98(2):1390–405. doi: 10.1021/acs.analchem.5c05327 (PMC12824988; doi:10.1021/acs.analchem.5c05327)
Supplement: Supplementary file 1 [file ac5c05327_si_001.pdf]

Supporting Information

Orthogonal investigation at single-particle and ensemble levels uncovers lipoprotein-extracellular vesicle binding

Angelo Musicò[1,2§], Roberto Frigerio[1,2,3§] Karl Normak[3], Sabrina Scolari[1] Alessandro Gori[2], Paolo Arosio[3], Annalisa Radeghieri[1,4], Lucia Paolini[4,5], Miriam Romano[4,6], Irantzu Llarena[7], Sergio E. Moya[7,8], Andrea Zendrini[1,3,4*], Paolo Bergese[1,4,9*]

[1] Department of Molecular and Translational Medicine (DMMT), University of Brescia, 25123 Brescia, Italy.

[2] Istituto di Scienze e Tecnologie Chimiche ‘‘Giulio Natta’’ – National Research Council of Italy (SCITEC-CNR), 20131 Milan, Italy.

[3] Department of Chemistry and Applied Biosciences, Institute for Chemical and Bioengineering, ETH Zürich, 8093 Zürich, Switzerland.

[4] Center for Colloid and Surface Science (CSGI), 50019 Sesto Fiorentino, Italy.

[5] Department of Medical and Surgical Specialties, Radiological Sciences and Public Health (DSMC), University of Brescia, 25123 Brescia, Italy.

[6] ASST Spedali Civili di Brescia, Dept. of Emergency, Spedali Civili University Hospital, Brescia, Italy.

[7] Soft Matter Nanotechnology, Center for Cooperative Research in Biomaterials (CIC biomaGUNE), Basque Research and Technology Alliance (BRTA), Donostia-San Sebastian, Spain.

[8] Faculty of Medicine, Chulalongkorn University, Bangkok, Thailand.

[9] National Inter-university Consortium of Materials Science and Technology (INSTM), 50121, Firenze, Italy.

§ These authors equally contributed to the work.

*Corresponding authors: andrea.zendrini@unibs.it, paolo.bergese@unibs.it,

Table of Contents

REVs and LPs fluorescent labelling and characterization S3

Calculation of the number of interacting LPs per REV S7

Determination of REV-LP interaction dynamics in solution using Fluorescence Cross-Correlation Spectroscopy S8

EV-LP binding stability and impact on EV membrane accessibility...S12

Label-Free immunodetection of EV-LP interaction...S13

DBSCAN clustering and colocalization maps S14

REVs and LPs fluorescent labelling and characterization

The fluorophores used in this study to label REVs and LPs for FCS, FCCS, SMR, and FC measurements were Atto NHS 633 and Atto DPPE 488, respectively. The emission and excitation spectra of the free fluorophores were analyzed using spectrofluorometry to confirm the absence of spectral overlap, ensuring accurate differentiation during subsequent analyses (Fig. S1A). FCS was employed to measure the diffusion coefficients of the free fluorophores, which were used to calibrate the instrument by defining the confocal volume. Additionally, FCS was used to characterize labeled NP preparations by determining the amount of free fluorophore. The diffusion coefficients of the fluorophores were 360 cm^2/sec for Atto DPPE 488 (Fig. S1B, green dot) and 340 cm^2/sec for Atto NHS 633 (Fig. S1B, red dot), corresponding to hydrodynamic diameters of 1.21 nm for Atto DPPE 488 (Fig. S1B, green triangle) and 1.29 nm for Atto NHS 633 (Fig. S1B, red triangle). REVs and LPs were labeled with Atto NHS 633 and Atto DPPE 488, respectively, following the protocols described in the Materials & Methods section. The fluorophore concentrations used for labeling were 250 nM for REVs and 1 µM for LPs. After overnight incubation, Atto NHS 633-labeled REVs and Atto DPPE 488-labeled LPs were characterized by DLS, fluorimetry, and FCS. Fluorescence emission spectra of the labeled NPs were acquired to optimize the detector range for FCS and FCCS measurements (Fig. S1C-D). Notably, the labeling process did not affect the emission spectra of the fluorophores.

DLS analyses showed no significant changes in the size distribution or the average hydrodynamic diameter (HD) of NPs due to the labeling process (Fig. S1E-F).

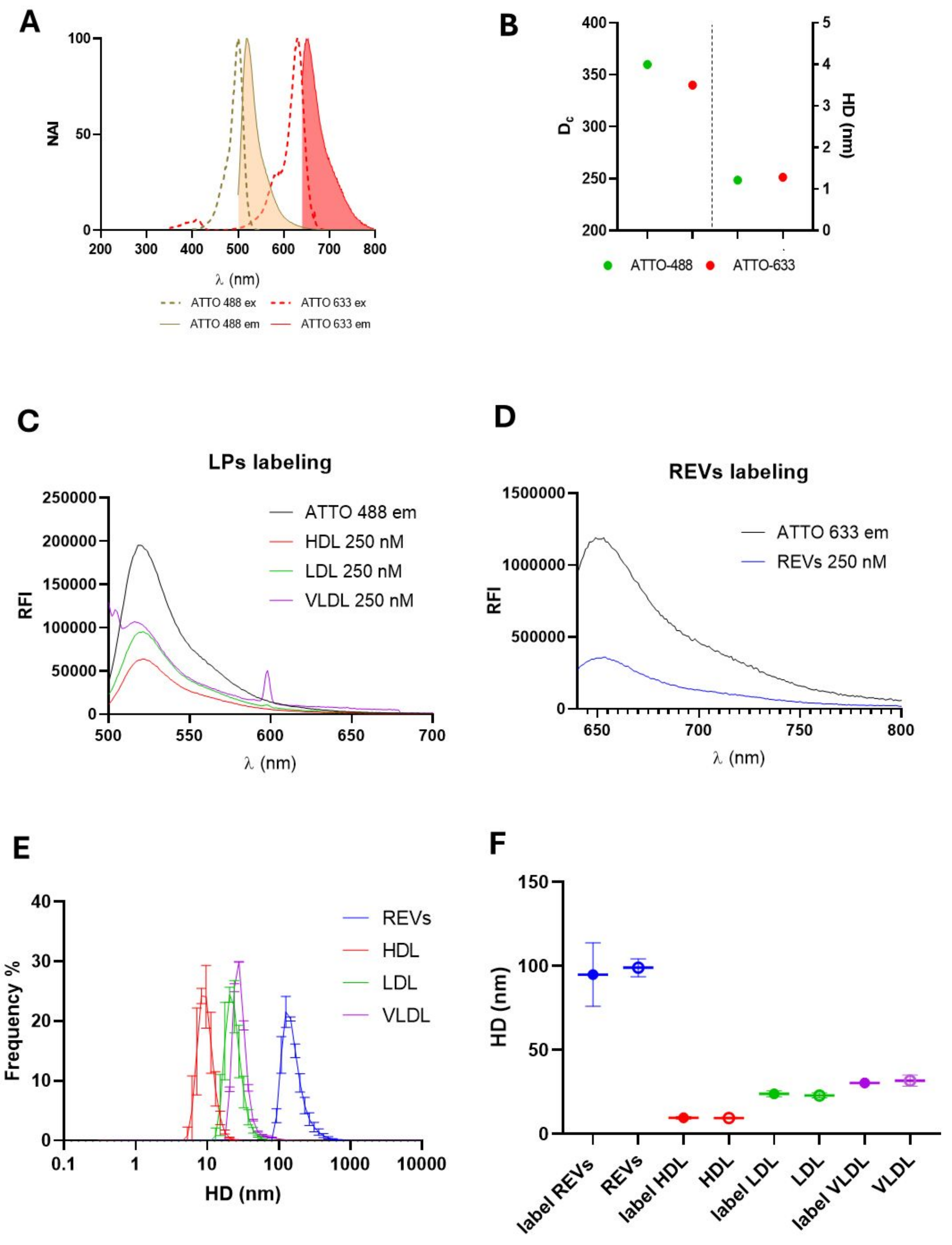

Figure S1: *A) Emission and excitation spectra graph, based on spectrofluorometer measurements. The analysis indicated that the two fluorophores have distinct and non-overlapping spectra. B) FCS measurements confirmed the diffusion coefficients of the two free fluorophores and their HDs. C) Emission spectra of labeled LPs. D) Emission spectra of labeled REVs. E) Size distribution of labeled NPs (REVs in blue, HDL in red, LDL in green, and VLDL in purple) measured by DLS. F) HD of*

NPs before and after the labeling protocol measured by DLS (full dots are used for labeled NPs, while empty dots are for unlabeled. REVs in blue, HDL in red, LDL in green, and VLDL in purple).

FCS was employed to further characterize the labeled nanoparticles. Figures S2A and S2B show the representative autocorrelation functions of REVs and LPs, respectively, compared with their corresponding fluorophore. The rightward shift of the function indicates an increase in the hydrodynamic diameter (HD) of the diffusing particles. The HD (Fig. S2C) and size distribution (Fig. S2D) measured by FCS are in good agreement with DLS measurements performed on both labeled and unlabeled samples. FCS characterization of REVs indicated nearly 100% labeling efficiency (Fig. S2E), meaning that all the Atto NHS 633 added to the REV preparation is bound to the EV surface. For LPs, FCS measurements showed labeling yields of 59% for HDL, 73% for LDL, and 99% for VLDL (Fig. S2E). Notably, due to the lipid nature of the DPPE probe, potential lipid exchange or "kissing" effects between NPs could impact labeling stability. Furthermore, we attempted to label LPs using different strategies (e.g., NHS ester coupling, as done for REVs) and added washing steps (via ultracentrifugation, size exclusion chromatography, and dialysis) to remove free fluorophore in the preparation. However, none of these strategies improved the labeling yield.

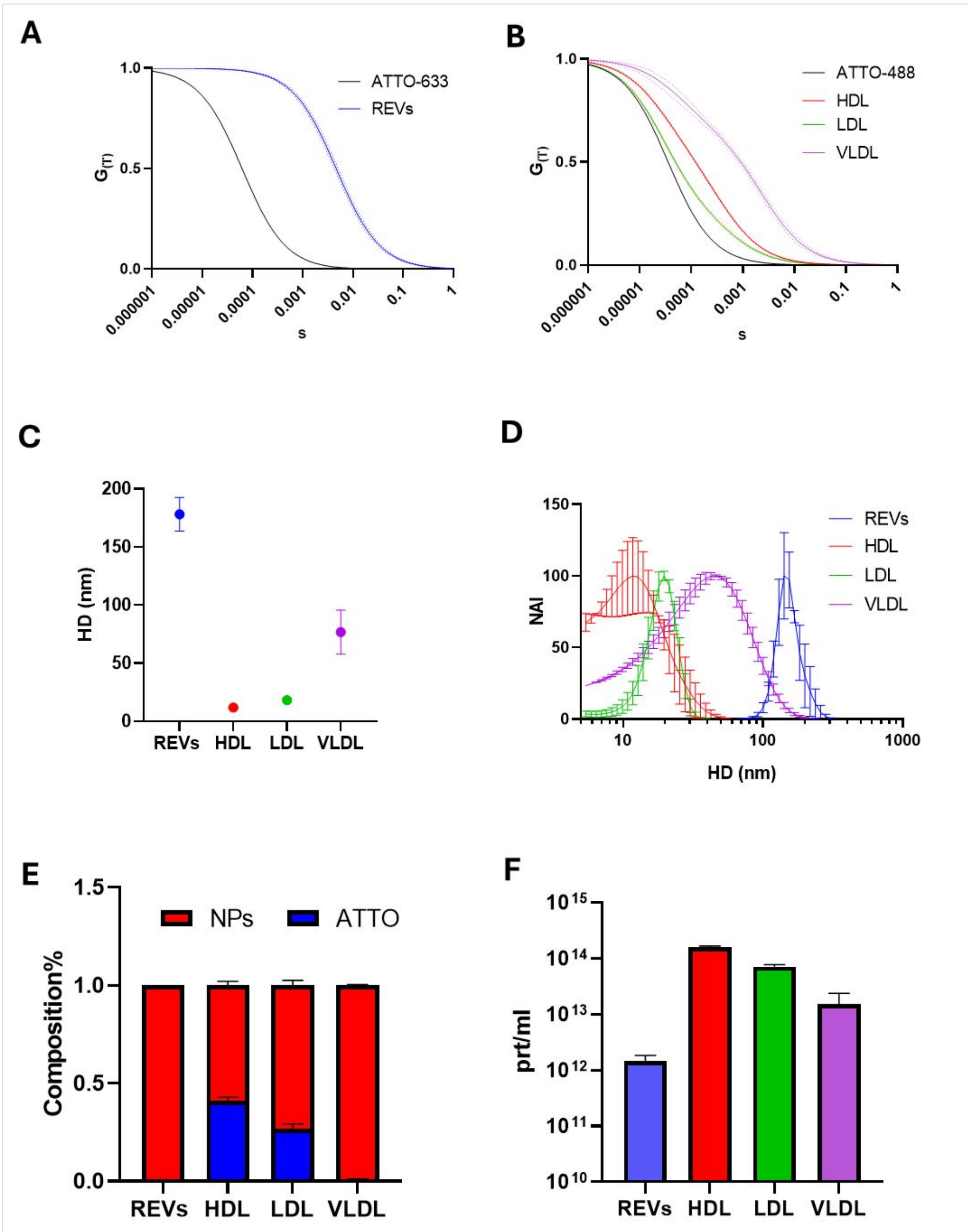

Figure S2: *A) Representative autocorrelation function of REVs (in blue) and ATTO-633 fluorophore (in black). B) Representative autocorrelation function of LPs (HDL in red, LDL in green, and VLDL in purple) and ATTO-488 fluorophore (in black). C) HD of NPs measured by FCS (REVs in blue, HDL in red, LDL in green, and VLDL in purple). D) Size distribution of NPs measured by FCS (REVs in blue, HDL in red, LDL in green, and VLDL in purple) determined by FCS. E) Labelling yields of NPs. The red portion represents labeled NPs, whereas the blue portion represents free fluorophores. The low labeling yield indicated significant contamination of free fluorophore and low labeling efficiency. F) prt/ml concentration of the NPs measured by FCS (REVs in blue, HDL in red, LDL in green, and VLDL in purple).*

Calculation of the number of interacting LPs per REV

Table S1 reports the particle counts of total, free and interacting LPs and REVs at t1 and t2 determined through FCCS.

t1	Total LPs	Free LPs	Interacting LPs	Interacting REVs	Total REVs
HDL	163.57 ± 6.70	113.51 ± 8.24	0.81 ± 0.11	0.93 ± 0.20	1.79 ± 0.25
LDL	60.19 ± 3.98	48.21 ± 2.38	0.69 ± 0.08	0.87 ± 0.13	1.77 ± 0.21
VLDL	5.18 ± 0.20	5.11 ± 0.04	0.07 ± 0.03	0.06 ± 0.02	1.75 ± 0.16
**t2**	**Total LPs**	**Free LPs**	**Interacting LPs**	**Interacting REVs**	**Total REVs**
HDL	160.27 ± 6.56	113.19 ± 7.16	1.56 ± 0.16	1.72 ± 0.20	1.74 ± 0.18
LDL	53.99 ± 4.48	44.21 ± 1.98	0.80 ± 0.18	1.07 ± 0.13	1.67 ± 0.15
VLDL	5.13 ± 0.2	5.01 ± 0.06	0.08 ± 0.03	0.05 ± 0.02	1.71 ± 0.20

Table S1. *Particle count of total, free, interacting LPs and REVs at t1 and t2 determined through FCCS.*

FCCS enables the identification of dual-colour events arising from LP–REV interactions. However, the technique does not provide information on the stoichiometry of each complex. Any dual-colour event is counted as a single diffusing species, regardless of how many LPs are actually bound to the same REV. As a consequence, the number of "interacting LPs" detected by FCCS corresponds to the number of interacting REVs, not to the number of LPs involved in the binding.

For these reasons, the stoichiometry of interaction between REVs and LPs has been calculated as follows (Eq. S1):

$$\frac{LPs}{REV} = \frac{(LPs_{total} - LPs_{free})}{LPs_{interacting}} \qquad \text{(S1)}$$

where:

- $\frac{LPs}{REV}$ is the number of LPs interacting per REVs
- LPs_{total} is the total number of LPs detected

- LPs_{free} is the number of unbound LPs
- $LPs_{interacting}$ is the number of interacting REVs, which corresponds to the number of interacting LPs measured

In this way, the calculation revealed that approximately 30 HDL particles interact per REV, compared to 10 for LDL and only 1 for VLDL, as shown in Figure S3.

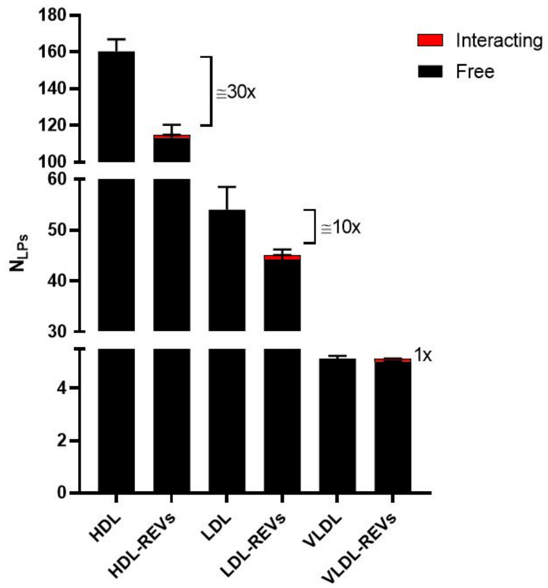

Figure S3. *Quantification of free and interacting lipoproteins (LPs). Black bars represent the number of free LPs measured when LPs are analyzed alone or after incubation with REVs at the same dilution. Red bars indicate the number of LPs detected in interaction with REVs. The brackets report the estimated number of LPs interacting per REV required to account for the observed decrease in the total count of free LPs for each LP class.* N_{lps}*: number of free LPs detected in the sample. REVs: fluorescent Red Blood Cell-derived EV sample. HDL: fluorescent HDL sample. LDL: fluorescent LDL sample. VLDL: fluorescent VLDL sample. HDL-REVs: fluorescent HDL-REV mixture sample. LDL-REVs: fluorescent LDL-REV mixture sample. VLDL-REV: fluorescent VLDL-REV mixture sample.*

Determination of REV-LP interaction dynamics in solution using Fluorescence Cross-Correlation Spectroscopy

FCCS measurements were performed in buffer (t1) and after the addition of plasma (t2) to evaluate the binding of REVs and LPs in two different environments. Figure S4 shows representative autocorrelation and cross-correlation functions for all the different samples measured. Specifically, Figure S4A presents data related to HDL-REV binding, Figure S4B shows LDL-REV binding, and

Figure S4C illustrates VLDL-REV binding. The Y-intercept for the autocorrelation functions is inversely proportional to the concentration of fluorescent objects, while the cross-correlation functions are directly proportional to the concentration of fluorescent objects.

Figure S4: *A) Representative autocorrelation function of REVs (in blue), HDL (in red), and their cross-correlation function (in black) at t1 (left panel) and t2 (right panel). B) Representative autocorrelation function of REVs (in blue), LDL (in green), and their cross-correlation function (in black) at t1 (left panel) and t2 (right panel). C) Representative autocorrelation function of REVs (in blue), VLDL (in purple), and their cross-correlation function (in black) at t1 (left panel) and t2 (right*

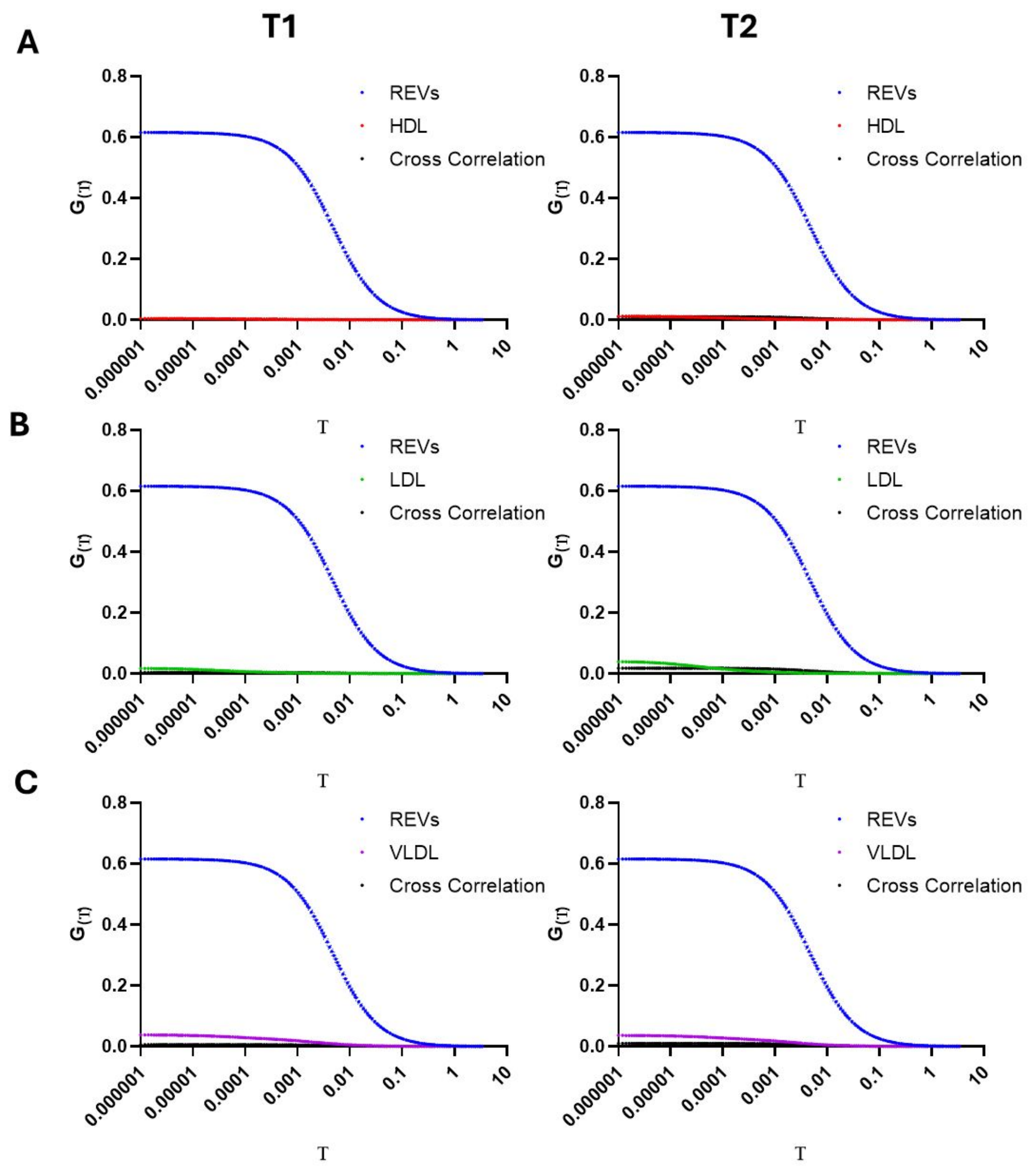

panel). REVs: fluorescent Red Blood Cell-derived EVs. HDL: fluorescent HDL. LDL: fluorescent LDL. VLDL: fluorescent VLDL. T: time.

We conducted dose-response experiments by incubating a fixed amount of REVs with increasing concentrations of LPs in both t1 and t2 (Figure S5). By analyzing the autocorrelation and cross-correlation functions, we calculated the fraction (ρ) of interacting REVs, which was plotted as a function of the LPs concentration. Data from the t1 dose-response experiments are shown in Figure S5A (HDL in red, LDL in green, and VLDL in purple). The dose-response curves were fitted using the Hill model to extrapolate the fitting parameters Bmax (Figure S5B), n (Figure S5C), and Kd (Figure S5D). Notably, for VLDL-REV binding at t1, we were unable to extrapolate the fitting parameters due to the weak binding observed. Interestingly, while LDL-REV binding patterns

remained similar between t1 and t2 (data from t2 are reported in the main text), HDL-REV binding exhibited significant changes. Specifically, there was an increase in the Kd and a reduction in n, suggesting that the environment plays a crucial role in modulating the binding of biomolecules to the EV surface.

Figure S5: *A) dose-response curve fitted with the Hill model of EVs-LPs interaction (red for HDL, green for LDL, and purple for VLDL) at t1. n = 3 B) Graphical representation of Bmax extrapolated from the fitting for each EV-LP interaction (red for HDL, green for LDL, and purple for VLDL). C) Graphical representation of n extrapolated from the fitting for each EV-LP interaction (red for HDL, green for LDL, and purple for VLDL), the dotted line represents n = 1. D) Graphical representation*

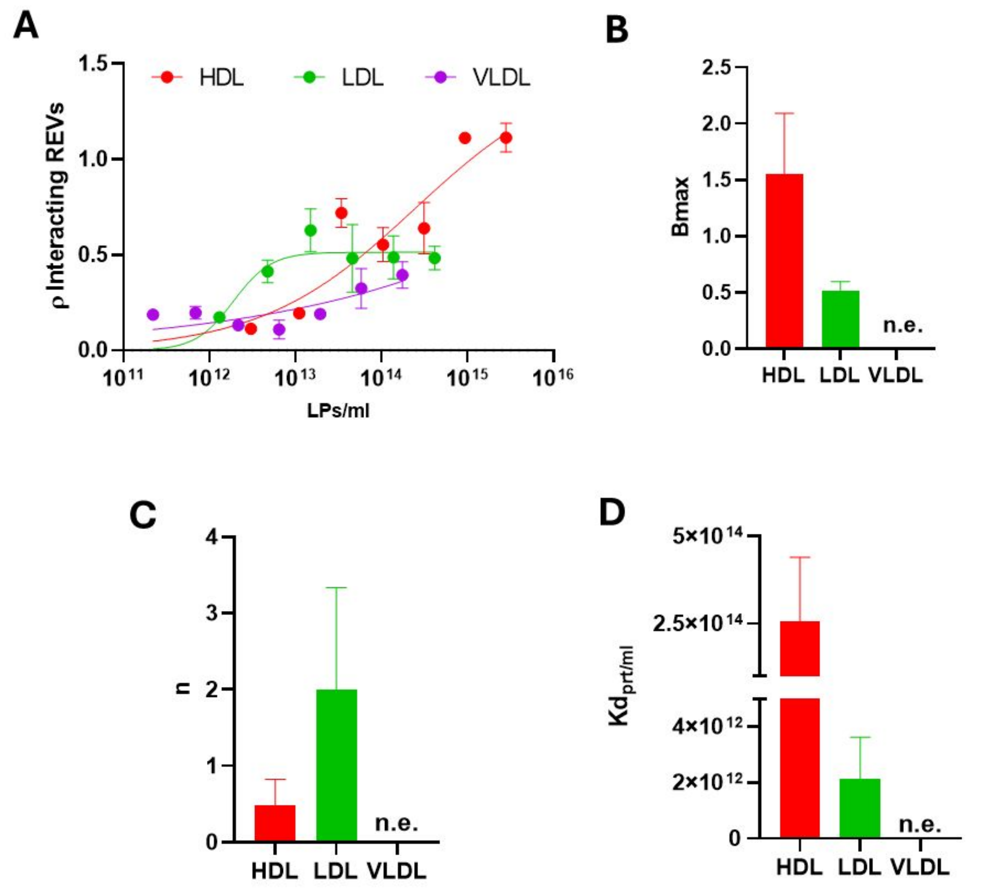

of Kd extrapolated from the fitting for each EV-LP interaction (red for HDL, green for LDL, and purple for VLDL). N.e.: extrapolation of the data is not allowed by the fitting model. REVs: fluorescent Red Blood Cell-derived EVs. HDL: fluorescent HDL. LDL: fluorescent LDL. VLDL: fluorescent VLDL.

EV-LP binding stability and impact on EV membrane accessibility

The REV fluorescence intensity signal (633 nm, red channel) was monitored across all LP concentrations (Figure S6). When using the same amount of REV, the signal remained constant at

lower LP concentrations. However, as the LP concentration increased, the signal gradually decreased. This trend suggests that at higher LP/REV ratios, LPs interact more extensively with the EV surface, reducing the available free surface area on the EVs. As a result, fewer binding sites remain accessible for the MSP.

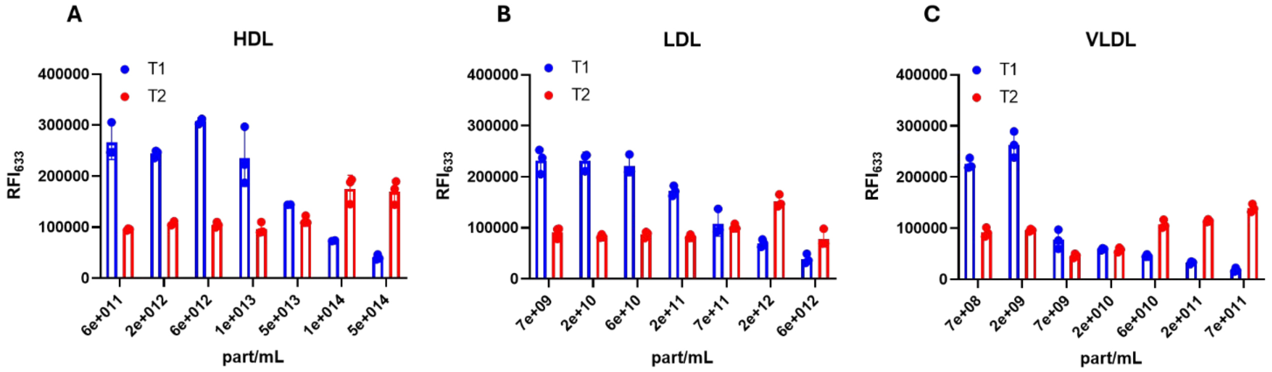

Figure S6: *the fluorescence intensity signal trends (633 nm, red channel) of REVs as a function of LP concentration. A) REVs fluorescence intensity at varying HDL concentrations (n = 3). B) REVs fluorescence intensity at different LDL concentrations (n = 3). C) REVs fluorescence intensity at varying VLDL concentrations (n = 3). HDL: fluorescent HDL. LDL: fluorescent LDL. VLDL: fluorescent VLDL.* RFI_{633}*: relative fluorescent intensity in the 633 channel (corresponding to REVs).*

We also monitored the fluorescence intensity signal for the LP channel (488 nm, blue channel) and observed distinct trends across all dose-response curves for LP samples (Figure S7). Specifically, in HDL, a similar pattern was detected under both t1 and t2 conditions. In LDL, a clear trend emerged at high LP concentrations, but at low LP-EV ratios, no distinct dose-dependent response was observed. In contrast, for VLDL, the signal was significantly reduced in t2 compared to t1, suggesting that plasma proteins negatively affect REV-VLDL adsorption. Additionally, in t1, the interaction initially followed a positive dose-response trend but dramatically decreased when the VLDL/REV ratio reached approximately 10:1.

Figure S7: *Fluorescence intensity signal in the LP channel (488 nm, green). A) Dose-response experiment showing fluorescence intensity at varying HDL concentrations with a fixed amount of REVs (n = 3). B) Dose-response experiment for different LDL concentrations with a fixed amount of REVs (n = 3). C) Dose-response experiment for varying VLDL concentrations with a fixed amount of*

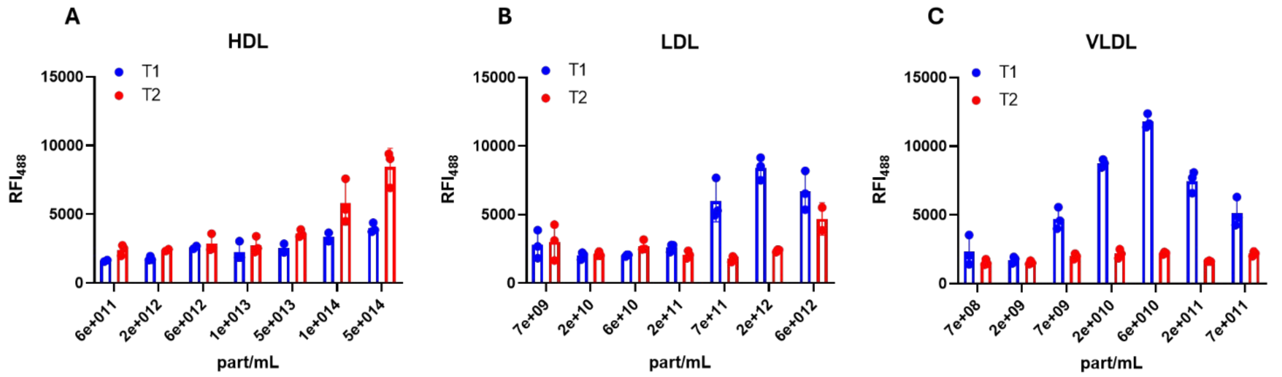

REVs (n = 3). HDL: fluorescent HDL. LDL: fluorescent LDL. VLDL: fluorescent VLDL. RFI_{488}: relative fluorescent intensity in the 488 channel (corresponding to LPs).

Label-Free immunodetection of EV-LP interaction

The results for the SiMoA assay performed at t1 are reported in Figure S8 and are in line with t2 results shown in the main text (Figure 8).

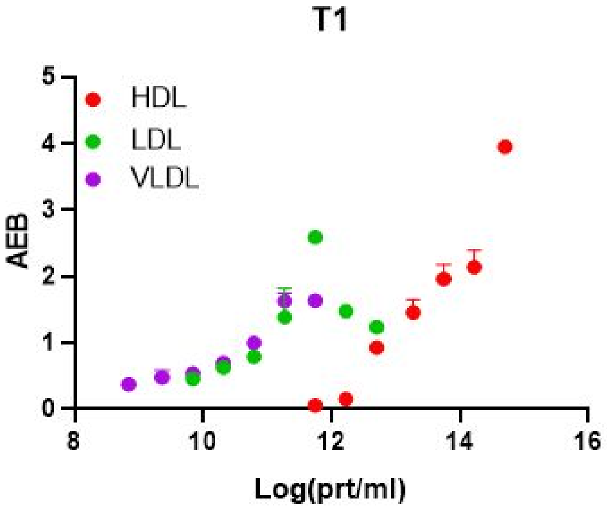

Figure S8: *Interaction of REVs and LPs at t1 (PBS) measured at different LP concentrations through immune hetero-sandwich SiMoA assay. Data are expressed in Average Enzyme per Bead (AEB) (n = 2). Legend: REV: unlabelled Red Blood Cell-derived EVs HDL: unlabelled High Density Lipoproteins. LDL: unlabelled Low Density Lipoproteins. VLDL: unlabelled Very Low Density Lipoproteins.*

DBSCAN clustering and colocalization maps

Representative DBSCAN clustering and colocalization maps of samples of REVs mixed with HDL, LDL and VLDL at t1 and t2 are reported in Figure S9.

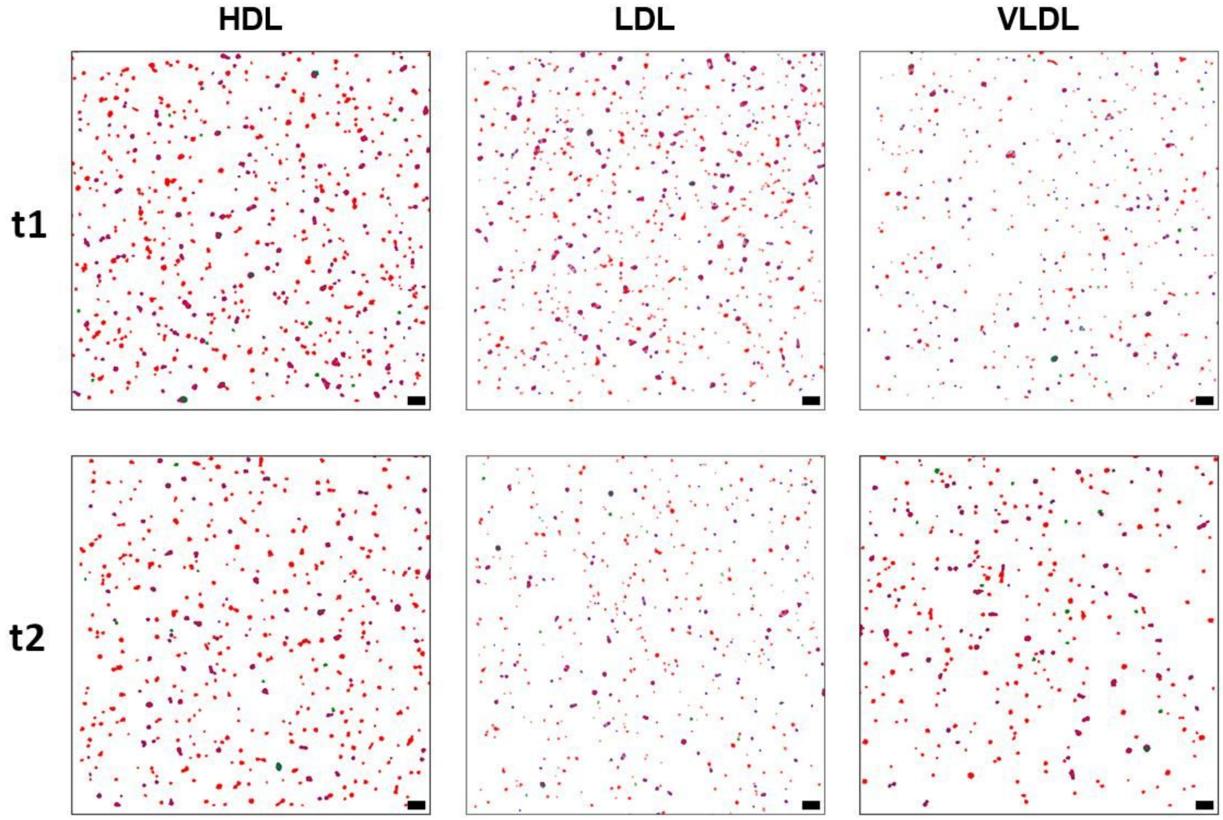

Figure S9. *DBSCAN clustering and colocalization maps of REV-LP samples at t1 and t2. Red clusters = REVs; Green clusters = LPs; blue outlines = colocalizing clusters. Scale bars 0.5 µm.*
